# Trio cooperates with Myh9 to regulate neural crest-derived craniofacial development

**DOI:** 10.7150/thno.51745

**Published:** 2021-02-25

**Authors:** Shuyu Guo, Li Meng, Haojie Liu, Lichan Yuan, Na Zhao, Jieli Ni, Yang Zhang, Jingjing Ben, Yi-Ping Li, Junqing Ma

**Affiliations:** 1 Jiangsu Key Laboratory of Oral Diseases, Nanjing Medical University, 140 Hanzhong Road, Nanjing 210029, China.; 2 Atherosclerosis Research Center, Key Laboratory of Cardiovascular Disease and Molecular Intervention, Nanjing Medical University, 140 Hanzhong Road, Nanjing 210029, China.; 3 Department of Pathology, University of Alabama at Birmingham, SHEL 810, 1825 University Boulevard, Birmingham, Alabama 35294-2182, USA.

**Keywords:** Neural crest cells, craniofacial deformity, Trio, Myh9, cell migration.

## Abstract

Trio is a unique member of the Rho-GEF family that has three catalytic domains and is vital for various cellular processes in both physiological and developmental settings. TRIO mutations in humans are involved in craniofacial abnormalities, in which patients present with mandibular retrusion. However, little is known about the molecular mechanisms of Trio in neural crest cell (NCC)-derived craniofacial development, and there is still a lack of direct evidence to assign a functional role to Trio in NCC-induced craniofacial abnormalities.

**Methods:*** In vivo*, we used zebrafish and NCC-specific knockout mouse models to investigate the phenotype and dynamics of NCC development in Trio morphants.* In vitro*, iTRAQ, GST pull-down assays, and proximity ligation assay (PLA) were used to explore the role of Trio and its potential downstream mediators in NCC migration and differentiation.

**Results:** In zebrafish and mouse models, disruption of Trio elicited a migration deficit and impaired the differentiation of NCC derivatives, leading to craniofacial growth deficiency and mandibular retrusion. Moreover, Trio positively regulated Myh9 expression and directly interacted with Myh9 to coregulate downstream cellular signaling in NCCs. We further demonstrated that disruption of Trio or Myh9 inhibited Rac1 and Cdc42 activity, specifically affecting the nuclear export of β-catenin and NCC polarization. Remarkably, craniofacial abnormalities caused by *trio* deficiency in zebrafish could be partially rescued by the injection of mRNA encoding *myh9*, ca-Rac1, or ca-Cdc42.

**Conclusions:** Here, we identified that Trio, interacting mostly with Myh9, acts as a key regulator of NCC migration and differentiation during craniofacial development. Our results indicate that *trio* morphant zebrafish and *Wnt1-cre;Trio^fl/fl^* mice offer potential model systems to facilitate the study of the pathogenic mechanisms of Trio mutations causing craniofacial abnormalities.

## Introduction

Neural crest cells (NCCs) are a group of cells found in vertebrate embryos with unique migration and differentiation abilities 1. Following epithelial to mesenchymal transition (EMT), NCCs migrate throughout the early embryo and differentiate into a wide array of cell types in the cranial region, including cartilage, the bones of the skull, teeth and connective tissues, as well as neurons and glia of the peripheral nervous system 2-4. Abnormal NCC migration and differentiation can cause many human diseases, such as craniofacial malformation 5-8.

Trio, also known as the triple functional domain protein, harbors 2 GEF domains and a protein serine kinase domain. The developmental roles of Trio have been extensively studied in recent years. Loss of Trio causes embryonic lethality along with abnormal development of skeletal muscle 9. Trio has also been implicated in multiple processes of neuronal development 10, including axon guidance, neurite outgrowth, and migration. Besides, Trio-mediated RhoA activation also plays an essential role during early eye development 11.

The functions of Trio in the dental-craniofacial complex are less established. Recently, studies have indicated that Trio is critical for NCC development. In *Xenopus*, Trio is expressed in migratory NCCs 12, and the Trio/Xcad-11 complex can regulate NCC migration 13. Mutations in TRIO may be involved in craniofacial abnormalities in patients who present with microcephaly and mandibular retrusion 14, 15. Despite the potential significance of Trio for NCCs, the direct evidence is insufficient to prove the functional role of Trio in NCC-derived craniofacial development.

Here, using morpholino knockdown of *trio*, we identify a required role for *trio* in the developing zebrafish, and we also generate NCC-specific knockout mice, which specifically target Trio in NCCs and their descendants in the target organs. We demonstrate that Trio is indispensable for NCC migration and differentiation and that Trio interacts extensively with Myh9 during embryogenesis. Furthermore, Trio deficiency decreases Rac1/Cdc42 activity in NCCs, which results in inhibited nuclear export and transcriptional activity of β-catenin and reduced NCC polarization. Our study represents a vital resource for understanding the molecular mechanisms involved in craniofacial development, which will have implications for future craniofacial abnormality treatments.

## Results

### Knockdown of *trio* in zebrafish leads to defects in NCC descendant differentiation

**Figure 1:**
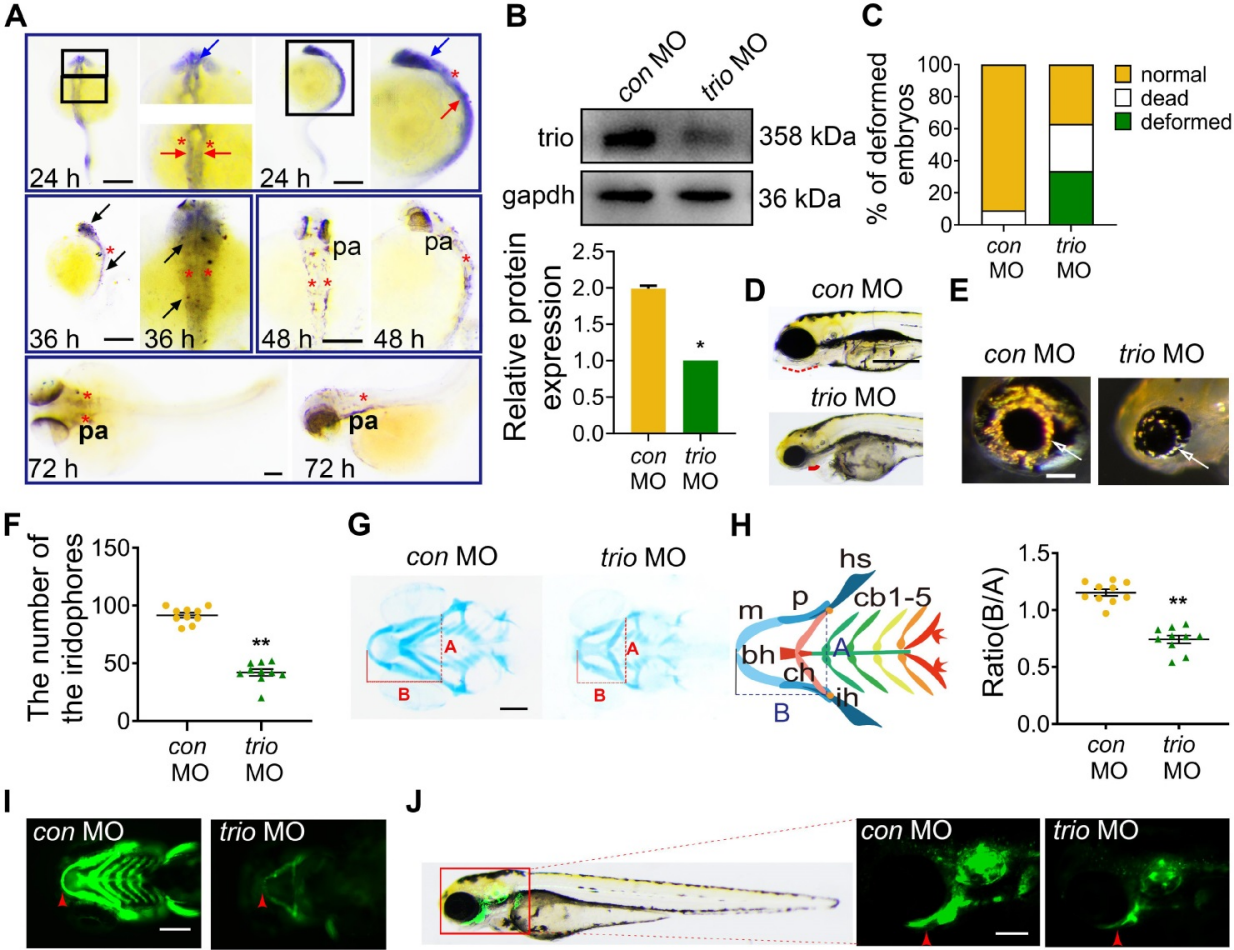
***trio* loss-of-function causes malformation of neural crest derivatives in zebrafish.** (A) WISH of *trio* in zebrafish embryos at 24, 36, 48 and 72 hpf. Bar = 100 μm. At 24 hpf, *trio* staining was detected in cranial NCC migrating around the eye (blue arrow) and developing ear (red asterisk), and in the NCC on either side of the neural tube (red arrow). Dorsal and lateral views of zebrafish at 36 hpf display prominent staining in the cranial and trunk NCCs (black arrow). Views of embryos at 48 and 72 hpf indicate *trio* expression around the pharyngeal arch tissues (pa). hpf: hours post-fertilization; WISH: whole-mount *in situ* hybridization. (B) Western blot showing the *trio* knockdown efficiency in the* trio* MO embryos, followed with quantification (n = 3). MO: morpholino. (C) Statistics of deformed, dead, and normal zebrafish embryos after the *trio* MO injection (n = 200). (D) Bright-field images of *con* MO embryos and *trio* MO embryos at 96 hpf. Red dotted line: mandible. Bar = 100 μm. (E) Images of eye iridophore (white arrow) at 96 hpf. Bar* =* 100 μm. (F) Quantification of eye iridophore amount and distribution at 96 hpf (n = 10). (G) Ventral view of *con* MO and* trio* MO embryos by Alcian blue staining at 120 hpf. Bar = 100 μm. Line A served as a baseline for later measurements and was used to normalize line B. Line B represents the distance from line A to the anterior of Meckel's cartilage. (H) Schematic of the ventral view of the pharyngeal arch cartilage structure in zebrafish embryos at 120 hpf. bh: basihyal; cb: ceratobranchial; ch: ceratohyal; hs: hyosymplectic; ih: interhyal; m: Meckel's cartilage; p: palatoquadrate. The quantification of the B/A ratio between *con* MO and *trio* MO embryos is shown. (I, J) Ventral (I) and lateral (J) views of 96 hpf Tg(*sox10:egfp*) *con* MO and* trio* MO zebrafish. Red arrowheads indicate mandible. Bar = 500 μm. For (F) and (H), data are represented as mean ± S.E.M. (two-tailed t test **p < 0.01).

We determined the temporal and spatial expression patterns of *trio* using whole-mount *in situ* hybridization (WISH) during the NCC migration stage of development and showed that at 24 hpf, *trio* was widely expressed in cranial NCC migrating around the eye (blue arrow) and developing ear (red asterisk), and in the NCC on either side of the neural tube (red arrow). At 36 hpf, *trio* expression was detected in the cranial and trunk NCCs (black arrow). At 48 and 72 hpf, the expression of *trio* was almost exclusively limited to the pharyngeal arch (pa) tissues (Figure 1A). To investigate the role of *trio* in NCC migration and development, we generated a *trio* knockdown zebrafish by injecting zebrafish embryos with an anti-sense morpholino (MO) against *trio* (*trio* MO) versus a standard *control* MO (*con* MO). After confirming a high *trio* knockdown efficiency using western blot (Figure 1B), we observed that embryos injected with *trio* MO appeared to have increased rates of mortality and curlier body shapes (Figure 1C, Figure S1A). Malformations were also detected in the *trio* MO group, including a retracted mandible, decreased iridophores at 96 hpf (Figure 1D-F, Figure S1B). The mandibular arch and iridophores are known NCC derivatives 16-18. At 120 hpf, Alcian blue staining of the craniofacial cartilage showed that Meckel's cartilage (i.e., the first pair of the cartilaginous pharyngeal arch, mandibular arch) was smaller in the *trio* MO embryos than in the control embryos, and the 3rd through 5th pairs were hardly detectable in the *trio* morphants (Figure 1G-H). Decreased ratios of B/A in trio morphants indicated that the position of Meckel's cartilage was posteriorly shifted, which may lead to mandibular retrognathism. Tg(*sox10:egfp*) zebrafish is a transgenic line expressing enhanced green fluorescent protein (EGFP) under the control of the *sox10* promoter, allowing EGFP-based detection of NCCs and their derivatives 19, 20. Fluorescence imaging of these animals confirmed the mandibular defects in the *trio* MO embryos (Figure 1I-J). Collectively, these results suggest that *trio* is indispensable for the normal development of NCC derivatives.

### Knockdown of *trio* suppresses the migration of NCCs in zebrafish

**Figure 2:**
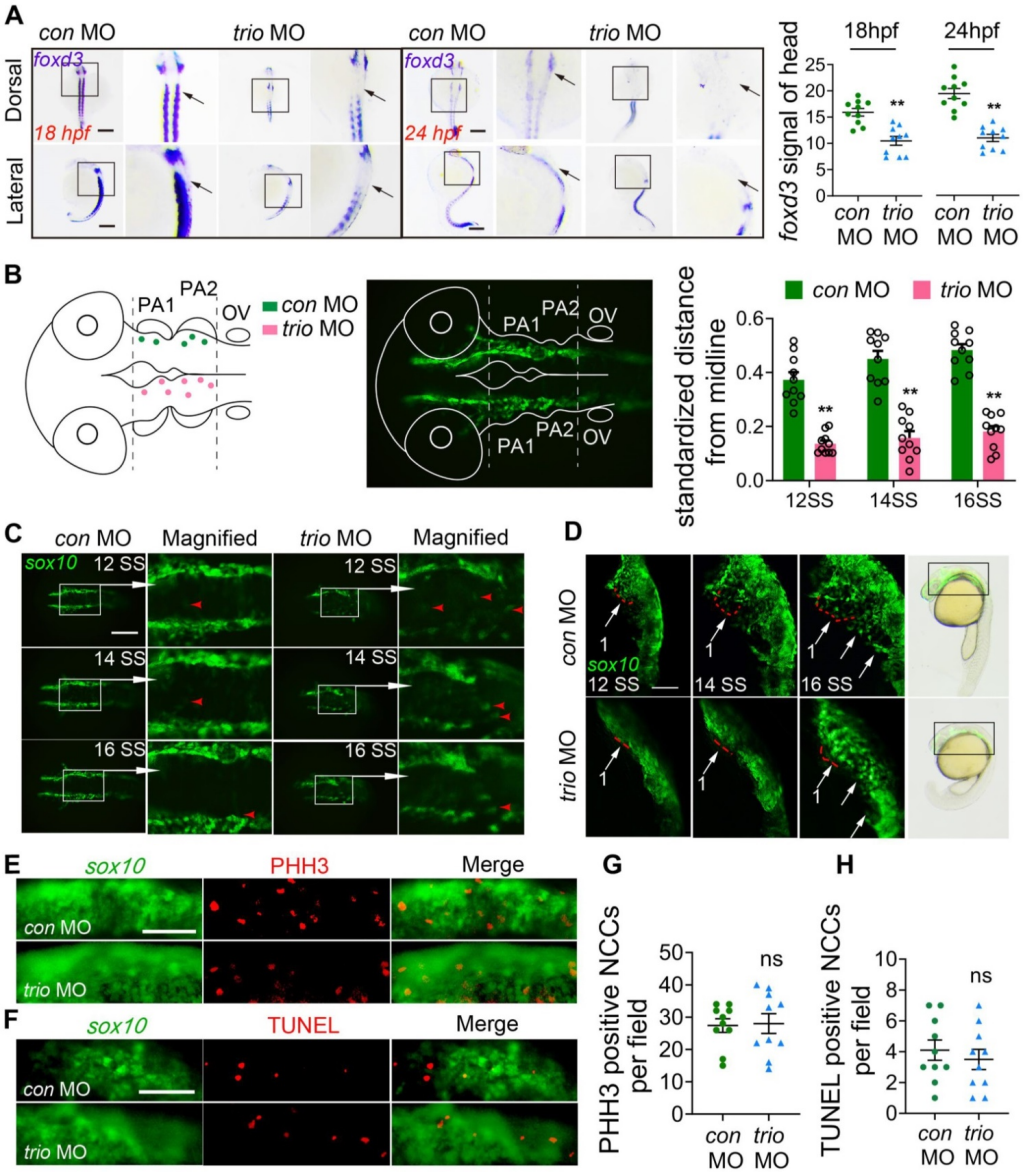
***trio* morphants show disruption of NCC migration.** (A) WISH was performed for the NCC migration marker *foxd3* in *con* MO and *trio* MO zebrafish embryos. The dorsal and lateral views of 18 hpf and 24 hpf embryos show *foxd3* expression in cranial NCC migratory streams (black arrow). Bar = 200 μm. Quantification of the *foxd3* mRNA signal detected in control or *trio* morphant embryos at 18 hpf or 24 hpf (n = 10). (B) Schematic of cranial NCC clusters distribution in the dorsal neural tube region surrounded by two dotted lines in the* con* MO and* trio* MO Tg*(sox10:egfp)* embryos from 12 SS to 16SS. The dots represent centroids of cell clusters, which are defined as two or more cells in contact with each other. Green dots above the midline represents *con* MO specimens, pink dots below the midline represents *trio* MO specimen. The middle schematic showed the study area in the fluorescence field. Normalized distances of centroids clusters from the midline in embryos at 12, 14, 16 SS were accounted for studying NCC migration tendency (n = 10). (C) Dorsal view of time-lapse images of *con* MO and *trio* MO Tg(*sox10:egfp*) live zebrafish embryos at 12, 14, and 16 SS. (The white frame highlights the NCCs in the area, while the red arrowhead denoting GFP-positive NCCs). The magnified images show NCCs in *trio* morphants appearing in the dorsal midline. Bar = 500 μm. NCCs: neural crest cells; SS: somite stage. (D) Lateral view of time-lapse images of *con* MO and *trio* MO Tg(*sox10:egfp*) embryos at 12, 14, 16 SS. The red dotted lines outline the first NCC migratory stream. The white arrows indicate the cranial NCC migratory streams. Bar = 250 μm. Bright field schematics show the location of green fluorescence at 21 hpf. MO: morpholino. (E) PHH3 staining of *con* MO and *trio* MO embryos indicates cranial NCC proliferation at 10 SS. Bar = 500 μm. PHH3: phosphohistone H3. (F) TUNEL staining of *con* MO and *trio* MO embryos indicates cranial NCC apoptosis at 10 SS. Bar = 500 μm. TUNEL: terminal deoxynucleotidyl transferase dUTP nick end labeling. (G, H) Quantitative analysis of PHH3 and TUNEL positive NCCs in the cranial region in (E) and (F). For (A), (B), (G) and (H), data are represented as mean ± S.E.M. (two-tailed t test **p < 0.01, ns, not significant).

The forkhead transcription factor *foxd3* and *crestin* are critical for neural crest development 21-23 and are expressed in premigratory and migrating cranial NCCs 24, 25. Using WISH, we detected *foxd3* expression in the cranial NCC migratory streams, which give rise to pharyngeal arches, in *con* MO embryos at 18 and 24 hpf. In contrast, *foxd3* expression was nearly absent in the cranial NCC migratory streams of the *trio* MO embryos and was significantly reduced in the third (branchial arch) stream (Figure 2A). Embryos at 18 hpf showed comparable *crestin* distribution in the cranial NCC migratory streams of the two groups (Figure S1F).

To investigate NCC behaviors in *trio* morphants, we also used the Tg(*sox10:egfp*) zebrafish model to visualize migratory NCC activity. We mapped the spatial localization of NCCs to display the unusual NCC clusters in *trio* MO embryos in more detail (Figure 2B). Dorsal view imaging and tracking of NCC movement from the 12-somite stage (SS) to the 16 SS were shown in Figure 2C. In Figure 2B, we also measured the distances between the NCCs and the midline, and expressed the distances as a ratio of the longest possible distance between any NCC and the midline at that specific anteroposterior position. The results showed that, in the controls, most of the EGFP-labeled NCCs were migrated ventrolaterally to the pa mesenchyme with the minority located lateral to the neural tube (Figure 2C). In the *trio* morphants, part of NCCs migrated out into the lateral pa mesenchyme, however, groups of closely-clumped NCCs (or NCC clusters) located just dorsal to the neural tube (Figure 2C). To confirm that NCC phenotypes shown in trio MO embryos at 12 SS-16 SS are not a delay in development secondarily caused by trio MO treatment, NCC migration was also investigated at more mature stages by the lateral view (Figure S1C). NCCs in the controls migrated in NCC migratory streams to ventrolateral regions, where they contributed to forming the pharyngeal arches and had normal cellular morphology. However, in the *trio* MO embryos, the NCC migratory streams had minimal migration from the ventral edge of the neural tube, and the cells clumped together amorphously (Figure 2D, Figure S1C, Movie S1). Besides, bright field images of zebrafish at 21 hpf showed that the length of the trio MO embryos body was not significantly different (Figure S1D). Together, the abnormal dorsal NCC clustering phenotype indicates that *trio* knockdown results in an inhibition of NCCs to undergo medial migration.

To see if abnormal cell proliferation or cell death causes NCC migration phenotypes, phosphohistone H3 (PHH3) immunostaining and terminal deoxynucleotidyl transferase dUTP nick end labeling (TUNEL) staining were performed in the Tg(*sox10:egfp*) zebrafish at 10 SS, before NCC migration phenotypes become apparent. At 10 SS, NCC migration was not affected in the *trio* morphant (Figure S1E). In the areas of pa mesenchyme and lateral neural tube, we found no significant changes in NCC proliferation and apoptosis in the *trio* morphants compared to the controls (Figure 2E-H). Furthermore, we also investigated whether *trio*-deficient NCC clusters might be failing to undergo medial migration because of abnormal proliferation and apoptosis in the dorsal neural tube. At 12 SS, controls and *trio* morphants also displayed comparable proliferation and apoptosis in the dorsal neural tube (Figure S1G-J), indicating that the formation of dorsal NCC clusters in the *trio* morphants is not related to NCCs undergoing abnormal proliferation and apoptosis.

Altogether, these results suggest that *trio* plays a critical role in NCC migration.

### Disruption of Trio causes defective craniofacial morphogenesis in conditional knockout mice

**Figure 3:**
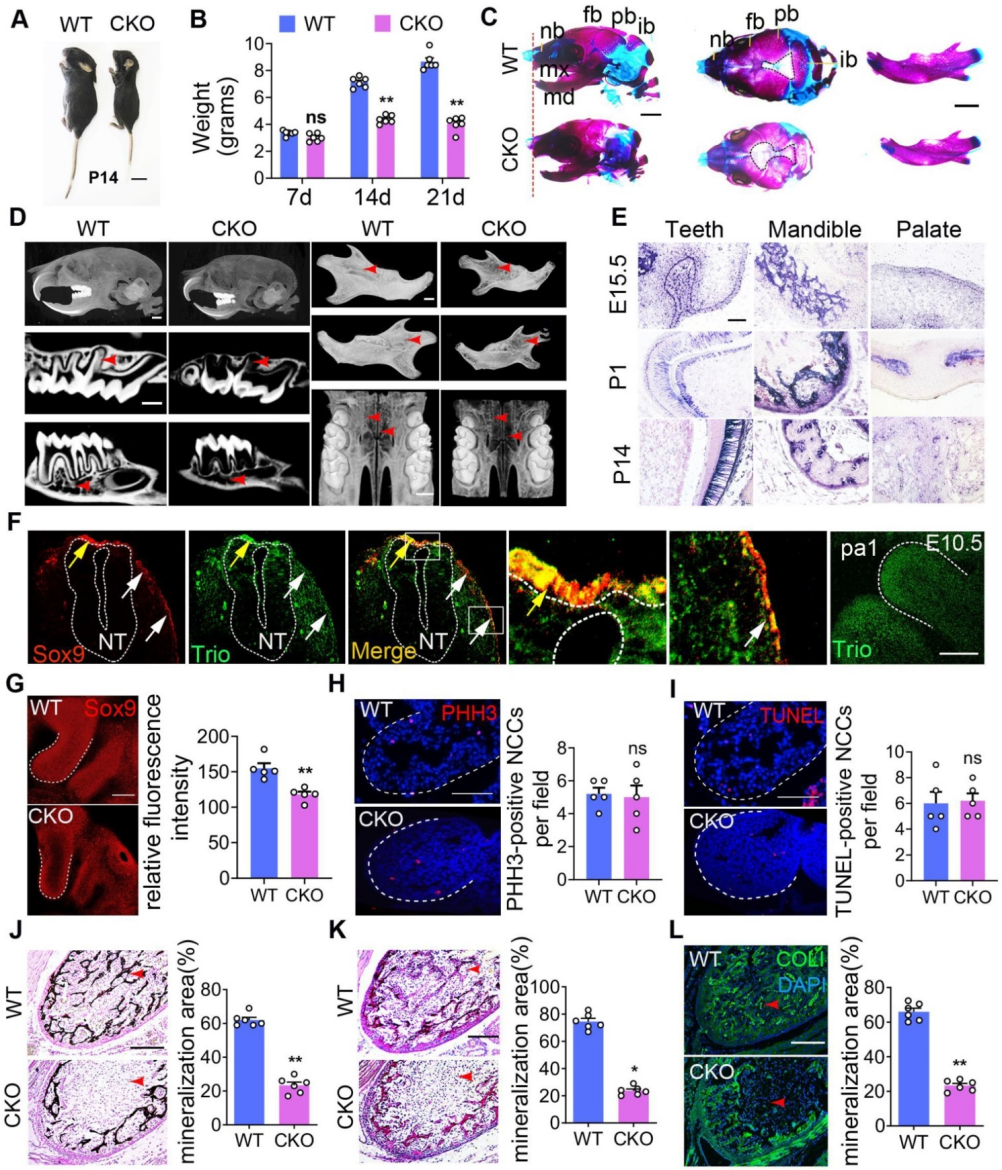
** Mice with Trio-deficient NCC-derived tissues show craniofacial defects.** Photograph of *Trio^fl/fl^*(WT) and *Wnt1-cre;Trio^fl/fl^*(CKO) mice at P14. The CKO image is representative of the smaller body size (see quantification in Figure S2B). Bar = 1 cm. P14, postnatal day 14. (B) Quantitative analysis of WT and CKO mouse weights at P7, P14, and P21 (n = 6). (C) WT and CKO mouse skulls and mandibles stained with Alizarin red and Alcian blue at P1. Bar = 500 μm. (D) Micro-CT image of the skull, teeth, mandible, and palate at P21. CKO mice display a significant reduction in mineralization (red arrowhead) of the skull, teeth, mandible, and palate. Bar = 500 μm. (E) WISH was performed for Trio expression in teeth, mandible, and palate at E15.5, P1, and P14. Blue-violet staining represents Trio anti-sense probe staining. Bar = 50 μm. (F) Immunofluorescence of Trio and Sox9 on sagittal sections of WT mouse embryos at E10.5. The colocalization (yellow) region demonstrates recombination in the premigratory (yellow arrow) and migratory (white arrow) NCC. Magnified images of the white dotted frame are attached to the right. NT: neural tube. The Far-right immunofluorescence of image is the sagittal section of WT mouse, which represents the primary expression of Trio in the pa1. Bar = 50 μm. (G) Immunofluorescence staining of Sox9 for cell migration in E9.5 CKO and WT embryos, with quantitative analysis (n = 5). Bar = 50 μm. (H) Immunofluorescence staining of PHH3 (red) for cell proliferation in sagittal sections through pa1 in E10.5 WT and CKO embryos. Bar = 50 μm. (I) Immunofluorescence staining with TUNEL assay (red) for cell death in sagittal sections through pa1 in E10.5 WT and CKO embryos. Bar = 50 μm. (J-L) Von Kossa (J), total collagen (K), and Collagen I (L) histologic staining of the mandible at P1, with quantification (n = 6). Red arrowhead: mineralization area. Bar = 100 μm. COL I, Collagen I. For (B), (G) - (L), data are represented as mean ± S.E.M. (two-tailed t test *p < 0.05, **p < 0.01, ns, not significant).

The advantages of the zebrafish model are multifactorial including their high genetic homology to humans, high fecundity, and ease of genetic manipulation. To develop an improved understanding of the human disease, we also used higher mammalian systems, such as conditional knockout mice. In the present study, compared to control (wild type, WT) mice, the *Wnt1-cre;Trio^fl/fl^* mice (CKO) mice were smaller in size and weighed less (Figure 3A, B, Figure S2A-B). Compared to the WT mice, the CKO mice also had a widened cranial frontal suture, mandibular retrusion and decreased mineralization in the skull, including the frontal, parietal, and interparietal bones and the alveolar and palate clefts in CKO mice (Figure 3C-D, Figure S2D-N), as determined by Alcian blue and Alizarin red staining of mice at postnatal day 1 (P1) and Micro-CT of mice at P21. The CKO mice showed more osteoporotic bone in the skull but had no change in the femur (Figure S2O-S). CKO mouse teeth were smaller than those of WT mice (Figure 3D, Figure S2C). We further examined the expression pattern of Trio in NCC-derived tissues using immunostaining and *in situ* hybridization analysis. At embryonic day 9.5 (E9.5), Sox9- or Trio- expressing cells (Figure 3F) were found in the dorsal neural tube (pre-migratory NCCs, yellow arrow), as well as outside of the neural tube (migratory NCCs, white arrows). At E10.5, Trio was widely expressed in the pharyngeal arch (Figure 3F), and it was also present at E15.5, P1, and P14 in the teeth, mandible and palate (Figure 3E, Figure S3A-B).

Given that the deletion of Trio in NCCs impaired craniofacial development, we hypothesized that Trio may have a vital effect on early NCC development in addition to its function in NCC migration. According to the references 26, 27, we performed immunostaining to detect SOX9, a marker of NCC migration, in the pharyngeal arch 1 (pa1) of E9.5 embryos. In Figure 3G, the CKO group displayed decreased NCC distribution in the pa1 by the staining of Sox9, suggesting defect in NCC immigration into the pa1 after ablation of Trio. Sagittal sections were immunostained with markers of cell proliferation (PHH3) and cell death (TUNEL) and were counterstained with DAPI. We found no significant change in proliferation and apoptosis between the two groups (Figure 3H-I)*.* Compared to the WT mice, the CKO mice at P1 had reduced mandibular mineralization as assessed by von Kossa (Figure 3J), total collagen (Figure 3K), and collagen I staining (Figure 3L). Therefore, these results indicate that Trio also has an important effect on the NCC developmental stage.

### NCCs lacking Trio show abnormal cell migration, differentiation and proliferation abilities *in vitro*

**Figure 4:**
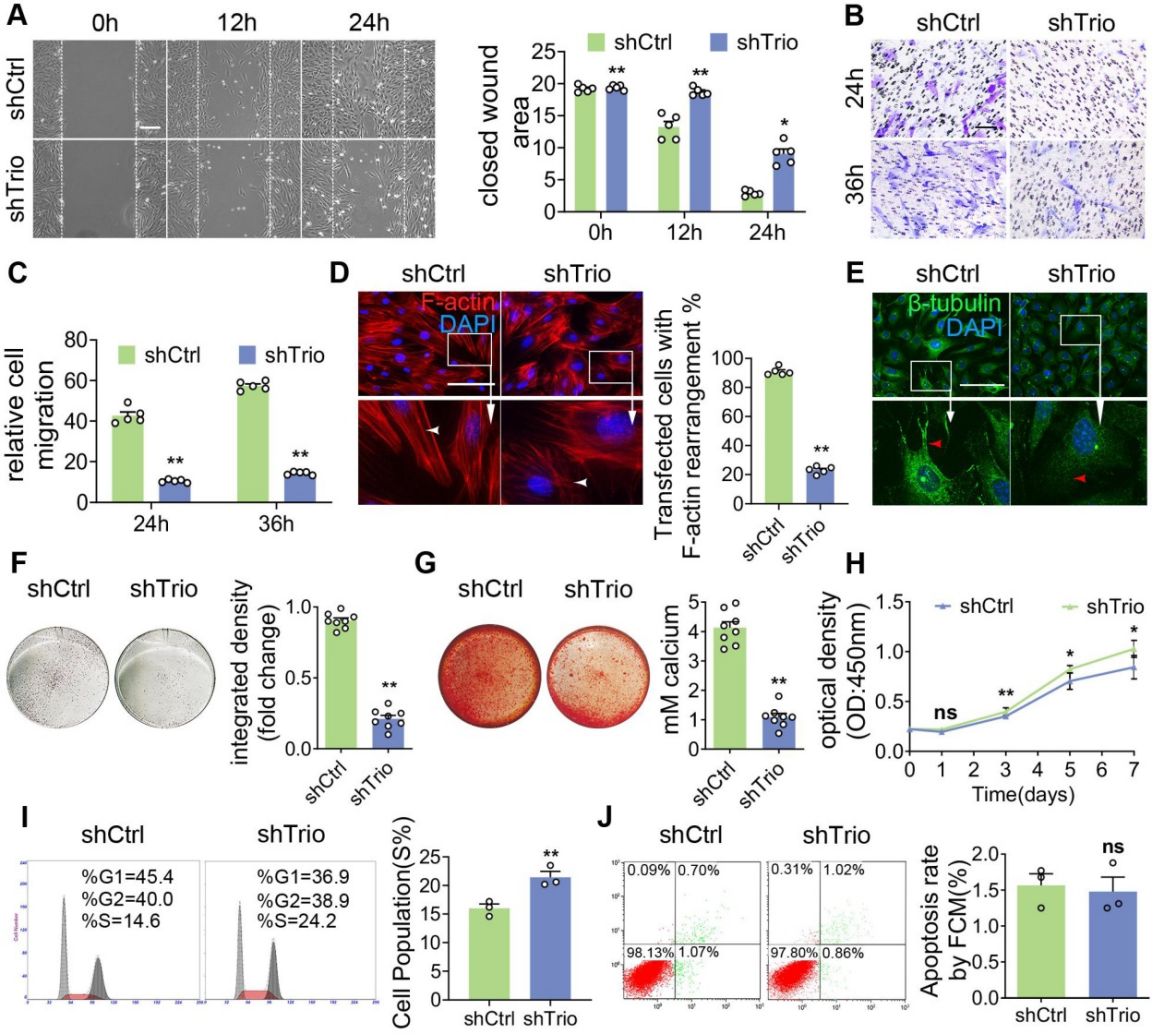
** Trio knockdown in NCCs negatively affects cell differentiation and migration *in vitro.***(A) Wound healing assays conducted and photographed at 0, 12, and 24 h, with quantification (n = 5). Bar = 200 μm. (B and C) Transwell migration assay of NCCs at 24 and 36 h after Trio knockdown, with quantification (n = 5). Bar = 100 μm. (D) Immunostaining of F-actin (red) in the cytoskeleton of shCtrl and shTrio NCCs, with quantification (n = 5). Nuclei were counterstained with DAPI (blue). Bar = 100 μm. DAPI: 4, 6-diamidino-2-phenylindole. (E) Immunostaining for β-tubulin (green) in the cytoskeleton of shCtrl and shTrio NCCs. The nuclei were counterstained with DAPI (blue). Bar = 100 μm. (F) Osteogenesis assessment using alkaline phosphatase (ALP) staining with quantification of NCC differentiation after five days of incubation in osteogenic medium (n = 8). Bar = 100 μm. shCtrl: short hairpin control (control lentivirus); shTrio: short hairpin Trio (Trio lentivirus). (G) Osteogenic differentiation assessment using Alizarin red staining (ARS) in NCCs after 14 days of incubation in osteogenic medium, with quantification (n = 8). Bar = 100 μm. (H) CCK8 assay at 0, 1, 3, 5, 7 days (n = 5). (I) Cell fractions of different phases detected by cytometry (n = 3). (J) Cell apoptosis measured by cytometry, with quantification analysis (n = 3). For (A) - (D) and (F) - (J), data are represented mean ± S.E.M. (two-tailed t test *p < 0.05, **p < 0.01, ns, not significant).

For an additional *in vitro* study, we knocked down Trio in primary NCCs (Figure S4A-D). To functionally evaluate the impact of Trio on migration ability, we used a wound healing assay and found that NCCs lacking Trio had reduced migration compared to controls (Figure 4A). Similarly, Trio knockdown cells showed a lower migratory capacity in a Transwell assay (Figure 4B-C). We evaluated the impact of Trio on the cellular organization using phalloidin staining, which showed that Trio knockdown cells had short, disordered actin filaments (Figure 4D) and more microtubule depolymerization (Figure 4E) than controls, suggesting that Trio is important for actin cytoskeleton organization and microtubule stability. These results demonstrate that Trio is required for NCC migration.

Then, we tested the osteogenic differentiation ability of NCCs in osteogenic media. NCCs lacking Trio had significantly decreased alkaline phosphatase (ALP) staining after 5 days of culture and Alizarin red staining after 14 days of culture (Figure 4F-G, Figure S4E-F), which indicated that Trio might promote NCC osteogenic differentiation. The cell cycle was determined by staining the cells with propidium iodide (PI) and performing flow cytometry, which, together with the CCK8 assay results, showed that cell proliferation was significantly increased in Trio knockdown NCCs (Figure 4H-I). However, no significant difference in apoptosis was found between the Trio knockdown NCCs and the NCCs without Trio knockdown (Figure 4J). Collectively, these results suggest that knockdown of Trio primarily impairs the osteogenic differentiation of NCCs, consistent with our *in vivo* observations in CKO mice. Taken together, these findings indicate that Trio elicits substantial effects in NCC migration, osteogenic differentiation and proliferation.

### Trio upregulates Myh9 expression and directly interacts with Myh9 in NCCs

**Figure 5:**
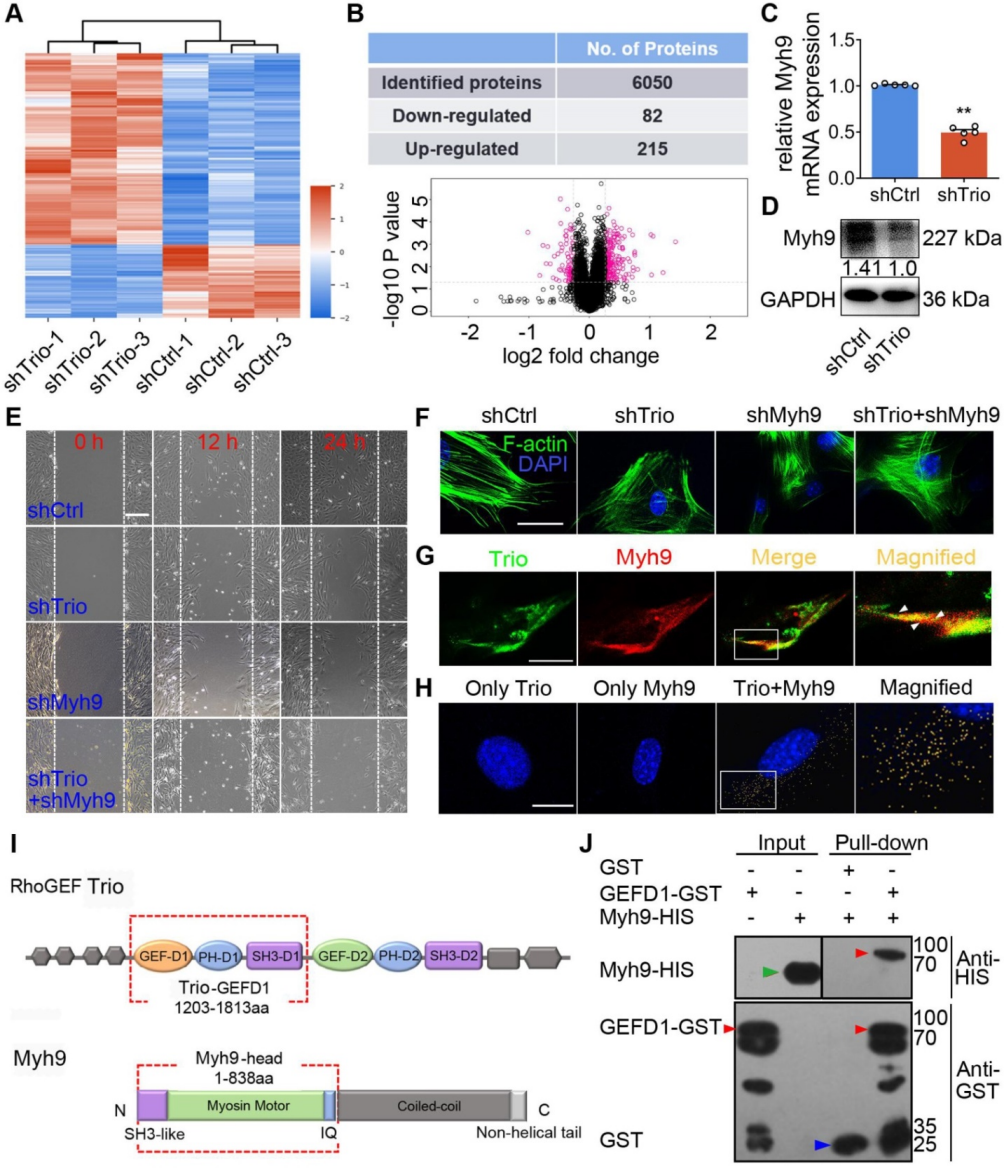
** Trio positively modulates Myh9 expression and physically interacts with Myh9 in NCCs.** (A) Heat map illustrating protein level changes following Trio knockdown in NCCs. The red and blue colors indicate higher and lower relative expression of proteins in shTrio compared to shCtrl, respectively. (B) Table quantifying the number of differentially expressed proteins (DEPs) and volcano plot demonstrating downregulated (red dots on the left) and upregulated proteins (red dots on the right) between the shCtrl and shTrio groups. (C) qRT-PCR of the Myh9 mRNA level in shCtrl and shTrio NCCs (n = 5). (D) Western blot of Myh9 protein expression between shCtrl and shTrio NCCs. (E) Wound healing analysis of the shCtrl, shTrio, shMyh9, and shTrio + shMyh9 groups at 0, 12 and 24 h. Bar = 200 μm. (F) Confocal microscopy images of F-actin (green) demonstrating NCC cytoskeleton morphology in the shCtrl, shTrio, shMyh9 and shTrio +shMyh9 groups. The nuclei were counterstained with DAPI (blue). Bar = 200 μm. (G) Colocalization (yellow) of immunostained Trio (green) and Myh9 (red). Bar = 200 μm. (H) PLA detection and visualization of Trio and Myh9 in NCCs. Blue: DAPI nuclear staining. Yellow spots: PLA signals. Bar = 400 μm. (I) Domain architecture of RhoGEF Trio and Myh9. Trio-GEFD1 and the Myh9 head domain shown in red frames highlight the binding domains. (J) Pull-down analysis of potential Trio and Myh9 interactions. Bound proteins were separated by SDS-PAGE in duplicate and analyzed by western blot with anti-HIS (Histidine) and anti-GST (Glutathione S-transferase) antibodies. The red arrow at 96 kDa indicates Trio-GEFD1, the green arrow at 68 kDa indicates Myh9, and the blue arrow at 25 kDa indicates GST.

To examine the potential mechanism underlying the function of Trio in NCCs, we performed proteomic analyses of NCCs to identify protein alterations associated with Trio knockdown by using isobaric tags for relative and absolute quantification (iTRAQ) (Figure 5A, Figure S5A). Of the 6050 proteins evaluated, 215 and 82 were significantly up- or downregulated, respectively, in Trio-deficient NCCs (Figure 5B). We analyzed the significantly altered genes using Gene Ontology (GO) and Kyoto Encyclopedia of Genes and Genomes (KEGG) pathway analyses (Figure S5B, E, Table S2-S4). We used the STRING database (http://www.string-db.org/) to search for protein-protein interaction networks (Figure S5C-D, F-G, Table S5) and, among the 82 (decreased) differentially expressed proteins (DEPs) in the shTrio NCCs, we identified a migration-related gene, Myh9, whose expression was substantially decreased. Real-time quantitative polymerase chain reaction (qRT-PCR) and western blot analysis (Figure 5C-D, Figure S5H) confirmed the reductions in Myh9 mRNA and protein in response to Trio inhibition in NCCs.

To confirm the effects of Myh9 on Trio-mediated NCC migration and differentiation, we transfected NCCs with Myh9 shRNA. A wound healing assay showed that Myh9 knockdown in NCCs further suppressed cell migration ability and worsened Trio-deficient NCC migration (Figure 5E). We observed a similar trend in F-actin cytoskeleton reorganization, as evidenced by phalloidin staining (Figure 5F). Moreover, NCCs lacking Myh9 had significantly decreased ALP staining after 5 days of culture and Alizarin red staining after 14 days of culture (Figure S6J-K), and worsened Trio-deficient NCC osteogenic differentiation in Trio and Myh9 double loss-of-function group (Figure S6J-K). After knockdown of Trio or Myh9, qRT-PCR analysis detected the mRNA expression levels of Alp, Runx2, Opn, Ocn, Osx, Col1a1, which are key genes involved in NCC osteogenic differentiation 28, 29. The mRNA expression levels of Alp, Opn, Ocn, Col1a1 decreased significantly in Trio or Myh9-deficient NCC (Figure S6L). Together, the results indicate that Myh9 may be involved in Trio-regulated NCC migration and osteogenic differentiation.

To determine the precise role of Trio and Myh9 during NCC development, it is important to assess the localization of the two proteins. Immunofluorescence staining showed Trio localization at the protrusions of NCCs and the plasma membrane at cell-cell contacts (Figure 5G), which was consistent with previous studies 5, 30. Interestingly, we observed that Trio and Myh9 were both found near cell-cell contacts and cell protrusions, suggesting an interaction between Trio and Myh9 (Figure 5G). In PLA assay, the use of only one primary antibody as a negative control led to no signal, validating the dual recognition requirement for *in situ* PLA assay signal generation. The PLA confirmed binding between Trio and Myh9, as evidenced by the multiple orange PLA spots indicating physical Trio-Myh9 interactions in NCCs (Figure 5H, Figure S5I). Using a GST pull-down assay, we identified the direct binding site between Trio-GEFD1 (amino acids 1203-1813) and the Myh9 head domain (amino acids 1-838) (Figure 5I). We further confirmed the interaction between these two proteins using a pull-down assay in which the Myh9 head domain was pulled down by Trio-GST (lane 4), indicating that the head domain of Myh9 contains the sequence responsible for Myh9 binding to Trio-GEFD1 (Figure 5J). Based on the knockdown phenotype, the subcellular localization of Trio and Myh9, and the interaction between these two proteins, we suggest that Trio and Myh9 are required for the formation of cell protrusions that are vital for NCC migration.

### Dissecting the functional roles of Trio and Myh9 in NCCs

**Figure 6:**
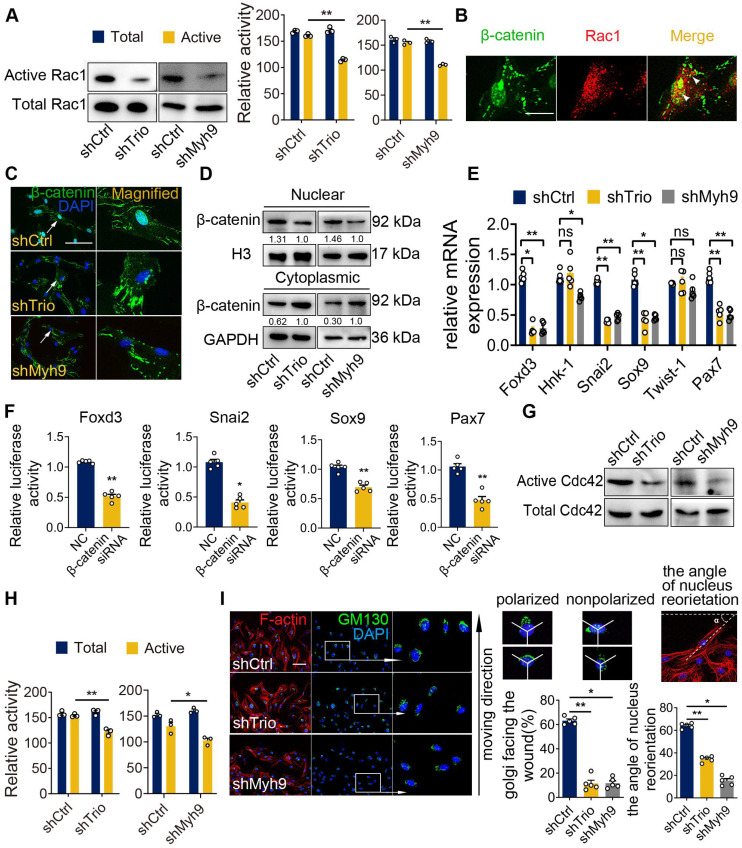
** Myh9 assists Trio in the regulation of NCC migration via Rac1 and Cdc42 GTPase activation.** (A) Rac1 pull-down activation in the shCtrl, shTrio, and shMyh9 NCC groups, with quantification (n = 3). (B) Immunostaining of β-catenin (green) and Rac1 (red) in NCCs. Bar = 100 μm. (C) Immunostaining of β-catenin (green) and DAPI (blue) for nuclear β-catenin expression indicated by a white arrow in shTrio and shMyh9 NCCs. Bar = 100 μm. (D) Western blot of β-catenin in the nucleus and cytoplasm in the shTrio and shMyh9 NCC groups. (E) qRT-PCR of migratory NCC marker genes (Foxd3, Hnk-1, Snai2, Sox9, Twist-1, Pax7) expression (n = 5). (F) Dual-luciferase reporter assay of relative NCC marker genes (Foxd3, Snai2, Sox9, Pax7) influenced by β-catenin (n = 5). (G and H) Cdc42 pull-down activation assay in the shTrio and shMyh9 NCC groups, with quantification (n = 3). (I) Immunofluorescence staining of the Golgi body marker GM130 and F-actin for polarization assay. The nuclei were counterstained with DAPI. The following schematic shows a polarized and nonpolarized cell, as well as the angle of nucleus reorientation. Bar = 100 μm. The quantification is shown as the percentage of Golgi facing the wound and the angle of nucleus reorientation (n = 5). For (A), (E), (F), (H), and (I), data are represented as mean ± S.E.M. (two-tailed t test *p < 0.05, **p < 0.01).

Because directional migration depends on the activation of small GTPases at the leading edge of cell protrusions and because Trio is a well-known GEF that likely acts upstream of the small GTPase family 31, 32, we evaluated whether Trio activated Rac1 and Cdc42 in NCC migration. By a conventional pull-down assay for the active form of Rac1, we detected that the level of Rac1 activation was decreased in Trio-deficient and Myh9-deficient NCCs (Figure 6A). β-catenin is a known downstream effector and binding partner of Rac1 33, 34 that localizes to the nucleus and regulates the transcription of multiple migration-associated genes, including Foxd3, Snai2, Sox9, and Pax7 35-37. Next, we investigated whether activated Rac1 could interact with β-catenin and affect the nuclear localization of β-catenin in NCCs. Using immunofluorescence staining, we found that Rac1 and β-catenin in control NCCs displayed partial colocalization (yellow spots) throughout the nucleus and cytoplasm, and the PLA assay confirmed this result (Figure 6B, Figure S6A). However, Trio-knockdown and Myh9-knockdown NCCs exhibited decreased transportation of β-catenin to the nucleus, which was further validated by western blot analysis of the nuclear and cytoplasmic protein levels of β-catenin (Figure 6C-D). The PLA assay showed that the colocalization of Rac1 and β-catenin in the nucleus of NCCs also decreased in the shTrio and shMyh9 groups (Figure S6B-C). To further explore whether β-catenin translocated into nuclei by Rac1 dependent manner, we used an inhibitor of Rac1-GTP (NSC23766) to inhibit Rac1 specific activation in NCCs (Figure S6D). Then, we found that NSC23766 inhibited activation of Rac1-GTP and decreased transportation of β-catenin to the nucleus by western blot analysis and immunofluorescence staining (Figure S6E-F). Together, these results indicate that Trio and Myh9 promote the nuclear localization of β-catenin by activating Rac1.

Foxd3, Snai2, Sox9, Pax7, Hnk-1 and Twist-1 are key genes involved in NCC migration 5, 38-40. qRT-PCR analysis showed that knockdown of Trio or Myh9 decreased the mRNA expression levels of Foxd3, Snai2, Sox9 and Pax7, however, the expression levels of Hnk-1 and Twist-1 had no significant change (Figure 6E). Immunofluorescence staining and western blot analysis further confirmed these results (Figure S6G-H). In the dual-luciferase assay, we constructed several reporter vectors, including Foxd3, Snai2, Sox9 and Pax7. Knockdown β-catenin inhibited the transcription of Foxd3, Snai2, Sox9 and Pax7 compared with that in the control group (Figure 6F). These findings indicate that Trio and Myh9 might promote β-catenin nuclear localization to initiate downstream gene transcription in NCCs by activating Rac1.

Cdc42 activation is considered a key modulator of cell polarity and adhesion 41, 42. First, we detected the active form of Cdc42 by a conventional pull-down assay, and the results showed that the level of Cdc42 activation was also decreased in Trio-deficient and Myh9-deficient NCCs (Figure 6G-H). To explore the effect of Trio on NCC polarity via Cdc42 activation, we examined the reorientation of the Golgi apparatus (GA) and nucleus in Trio-knockdown and Myh9-knockdown NCCs as a method to determine the directional migration of the cells 43-45. After 12 h of migration in a wound healing assay, we stained NCCs for the Golgi marker GM130, and the nucleus was stained with DAPI. In response to the wound, the NCCs in the two groups reoriented their Golgi bodies and nuclei (Figure 6I). However, there was a distinct reduction in the number of cells exhibiting GA polarization and nuclear reorientation in the shTrio and shMyh9 groups (Figure 6I). Moreover, when Trio-deficient NCCs were briefly stimulated with a commercial Cdc42 activator (1 U/mL), a dramatic increase in Golgi nuclear reorientation occurred (Figure S6I).

Collectively, these results suggest that Trio and Myh9 may drive NCC migration via Rac1 and Cdc42 GTPase activation.

### *myh9* and ca-Rac1/ca-Cdc42 mRNA partly restore the defects in *trio* morphants

**Figure 7:**
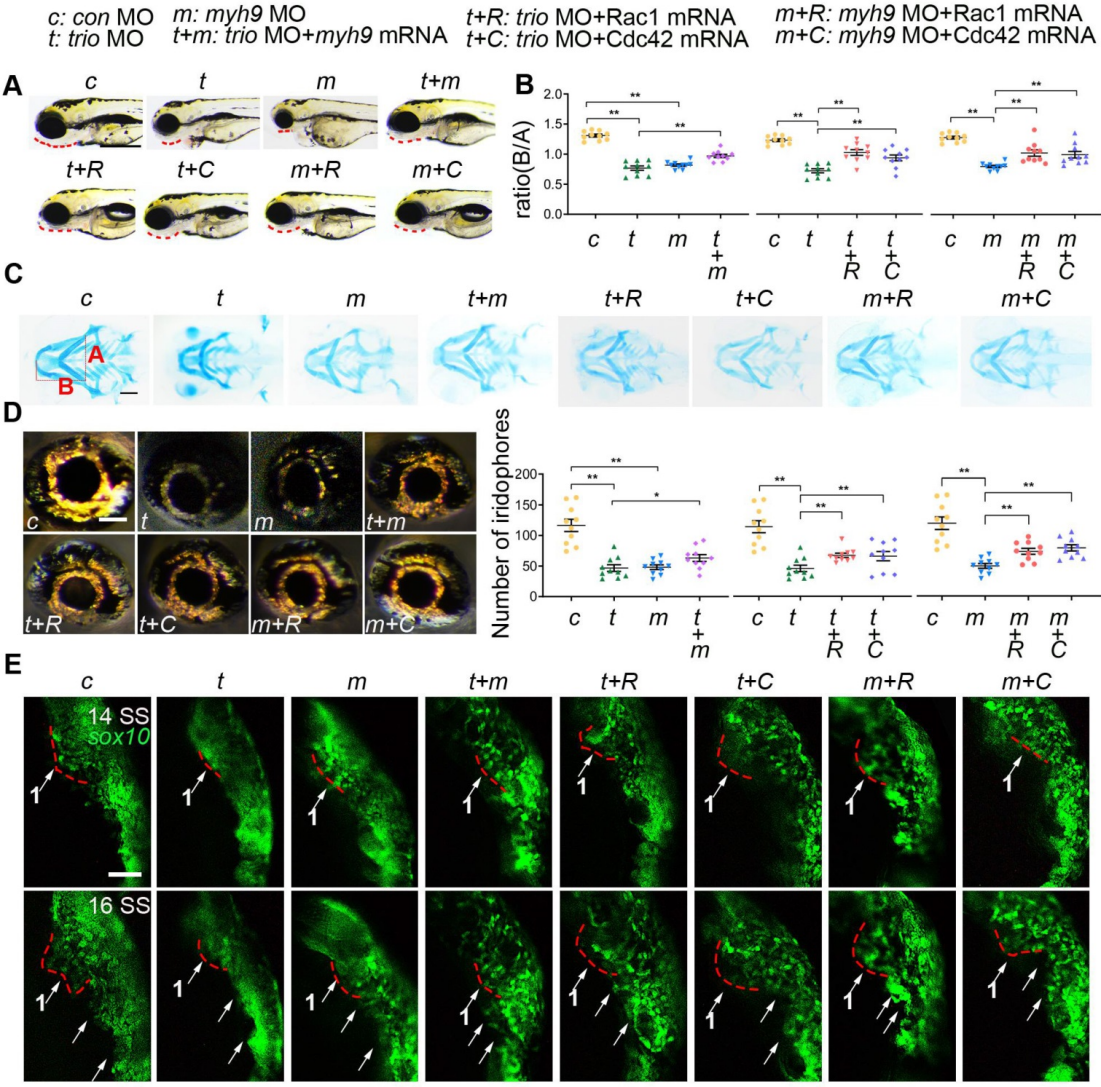
***myh9* and ca-Rac1/ca-Cdc42 partly restore the defects in *trio* morphants.** (A) Bright field image of zebrafish embryos at 96 hpf. Bar = 100 μm. Red dotted line: mandible. “+”, coinjection. MO, morpholino. hpf, hours post-fertilization. *c*: con MO, *t*: *trio* MO, *m*: *myh9* MO, *t+m*: *trio* MO+*myh9* mRNA, *t+R*: *trio* MO+Rac1 mRNA, *t+C*: *trio* MO+Cdc42 mRNA, *m+R*: *myh9* MO+Rac1 mRNA, *m+C*: *myh9* MO+Cdc42 mRNA. (B, C) Images and quantification of Alcian blue-stained zebrafish embryos at 120 hpf (n = 10). Bar = 500 μm. (D) Eye iridophore amount and distribution of the experimental groups (n = 10). Bar = 100 μm. (E) Time-lapse images of live Tg(*sox10:egfp*) zebrafish at 14 and 16 SS in the experimental groups mentioned above. Bar = 100 μm. SS, somite stage. For (B) and (D), data are represented as mean ± S.E.M. (two-tailed t test *p < 0.05, **p < 0.01).

Because of the effect of Trio on neural crest migration and differentiation and its direct interaction with Myh9 in NCCs, we wanted to further validate the results by performing a rescue assay in a zebrafish model. We established *myh9* knockdown zebrafish embryos by injecting anti-sense morpholino against *myh9* (*myh9* MO) and designed mRNA encoding *myh9* and the constitutively active form of Rac1/Cdc42 (ca*-*Rac1, ca*-*Cdc42) to overexpress *myh9* and constitutively active Rac1/Cdc42. The phenotype of zebrafish in the *myh9* MO group displayed a retrusive mandible at 96 hpf, a posterior shift of Meckel's cartilage at 120 hpf (Figure 7A-C), and decreased iridophores at 96 hpf (Figure 7D), which was similar to the *trio* morphant group. However, coinjection of *trio* MO and *myh9* mRNA partially rescued the defects caused by *trio* deficiency (Figure 7A-D). Additionally, partially restored mandible development and eye iridophores were also detected in embryos coinjected with *trio* MO and ca*-*Rac1/ca*-*Cdc42 mRNA (Figure 7A-D). Importantly, ca*-*Rac1 and ca*-*Cdc42 mRNA were also able to rescue the phenotype in the *myh9* MO-injected embryos (Figure 7 A-D).

We also explored the role of *trio* and *myh9* in neural crest migration using Tg(*sox10:egfp*) zebrafish. Time-lapse images showed that in *trio* and *myh9* MO embryos, the cranial NCC migratory streams from the ventral edge of the neural tube were inhibited, and the cells clumped together amorphously (Figure 7E). Time-lapse images showed that the NCCs of *trio* MO embryos injected with *myh9* mRNA had increased long-range migration away from the medial neural tube in *trio* MO embryos from the 14 SS and 16 SS. The red dotted line denotes the first NCC migratory stream (Figure 7E). To confirm whether the phenotypes in the *trio* and* myh9* MO groups were induced by inhibited *rac1* and *cdc42* GTPase activation, the *trio* and* myh9* MO embryos were rescued by the injection of ca*-*Rac1 or ca*-*Cdc42 mRNA. The results showed that the *trio* and* myh9* MO embryos injected with ca*-*Rac1 or ca*-*Cdc42 mRNA exhibited significantly increased long-range migration away from the medial neural tube compared with the mutant group, but this process was still slightly slower than that of the WT group injected with the *con* MO (Figure 7E). This partial rescue confirms our* in vitro* data showing that Trio regulates NCC migration and craniofacial development via Rac1 and Cdc42 signaling and that Myh9 functions as a binding partner of Trio to enhance this process.

## Discussion

Trio is primarily known for its role in neuronal development 46, 47, and its physiological function in craniofacial development has not been fully investigated. Understanding the pathogenetic factors of craniofacial diseases and the molecular mechanisms governing craniofacial development will benefit the search for possible therapeutics. In this research, we demonstrated the function of the RhoGEF Trio in NCC development and revealed that it is required for the migration of NCCs as well as the development of the craniofacial bones. In this pathway, Myh9 functions as a binding partner of Trio-GEFD1 that cooperatively strengthens the role of Trio in regulating NCC development and craniofacial development (Graphical Abstract).

Both Trio and Myh9 have been reported to control cell migration; however, the functional interaction of the two proteins in NCC migration remains elusive. Myh9 is part of the myosin II subfamily and is expressed widely in early embryos 48. Myh9 is uniquely responsible for cell contractility 49 as well as directional cell migration 49, 50. Complete depletion of Myh9 in mice is lethal 50, and mutations in MYH9 are associated with susceptibility to developing craniofacial deformities in humans 51. These data suggest that Myh9 may be involved in NCC migration and craniofacial development. Similar to Myh9, Trio has also been implicated in the regulation of NCC migration. Previous studies have shown that Trio interacts with cadherin 11 to regulate filopodia and lamellipodia formation in Xenopus NCCs 13. In addition, the Wnt signaling molecule Dishevelled (DVL) forms a complex with Trio to activate Rac1, which participates in promoting Xenopus NCC migration 30. In our study, we showed the functional and physical interaction of Trio with Myh9. First, we showed that both proteins interacted in NCCs via the head domain of Myh9 and the GEFD1 domain of Trio. Second, this interaction likely took place in the cell-cell contacts and cell protrusions, which indicated that directional NCC migration depends on the interaction of these two proteins. Furthermore, Myh9 knockdown exacerbated aberrant migration in the context of Trio deficiency in NCCs, and coinjection of *myh9*, ca-Rac1 or ca-Cdc42 mRNA rescued *trio* knockdown in zebrafish. Taken together, these data suggest that Trio promotes NCC migration by interacting with Myh9. This leads to the activation of Rac1 and Cdc42, but other yet unknown signaling mechanisms may also participate in this process.

In addition to these firmly established roles in NCC migration, Trio is also vital for NCC differentiation. The role of Trio in osteogenic differentiation has not been previously reported. In our study, Trio activity was essential for osteogenesis and thus for the formation of the craniofacial bones originating from the NCCs, and the mesoderm-derived femur bones were not affected (Figure S2O-S). However, our* in vivo* findings showed that the mineralization of non-NCC-derived craniofacial bones, like parietal bones, also decreased significantly in the CKO mice (Figure S2J-N). This may be caused by defective sagittal suture development. The sagittal suture, a kind of fibrous connective tissue, connects the frontal, parietal, and interparietal bones (Figure S2I). Cells in the suture tissue are derived from NCCs 52 and differentiate into osteoblasts, which deposit collagen fibers and increase the mineralization of the surrounding bones 4, 53, 54. Trio knockout in NCCs may disrupt the normal process of sagittal suture development, which results in decreased mineralization of surrounding bones, including non-NCC-derived bones. Moreover, mesoderm-derived cells are surrounded by NCCs, and this complicated arrangement has an important significance for craniofacial development. Studies have also demonstrated that in the coordinated morphogenetic program with the mesoderm, NCCs may act as the conductor by sending signals to guide the differentiation of mesodermal cells 55-57. The specific mechanisms of Trio-regulated NCC osteogenic differentiation and the seemingly paradoxiåcal results of decreased mineralization, including of non-NCC-derived bones, need to be further investigated in future studies.

In the present study, for the effect of Trio on the proliferation of NCCs, the data from *in vivo* and* in vitro* experiments are inconsistent. Cell behavior and function are determined through the constant interactions with multiple factors within their complex cell micro-environment. *In vitro* assay lays the foundations for studies of cell functions and relative mechanisms, however, it fails to rebuild the intricate environment of a cell, a tissue or ultimately an organism *in vivo*. This may explain the discrepancy between the proliferation results of* in vivo* and *in vitro* assays.

β-catenin is a key component of canonical Wnt signaling, and conditional knockout β-catenin in the NCCs is known to increase apoptosis of migrating NCCs, leading to severe craniofacial malformations 58. Study also showed that disrupting Fas-associated factor 1, one of the main sensors of the extrinsic apoptosis pathway, exhibited increased cytosolic β-catenin accumulation and apoptosis resistance 59. Although the previous study indicated that NCCs deleting β-catenin led to elevated apoptosis, our data showed no significant difference of apoptosis after Trio knockdown in NCCs accompanied by decreased nuclear and increased cytoplasmic β-catenin protein, suggesting the different role of β-catenin in cytoplasm and nuclei to regulate NCC apoptosis. The specific mechanisms of β-catenin in NCC apoptosis need to be further investigated in future studies.

Our study revealed short root abnormalities in CKO mice, and Trio has been proven to be a coordinator in regulating mouse tooth development 60. Consistent with this, we found that human stem cells of the dental papilla (SCAPs) from short root teeth exhibited reduced TRIO expression compared with those of normal teeth (Figure S7). A prior study by Wei *et al*. sequenced TRIO in >2300 patients using molecular inversion probes and found that individuals with mutations in TRIO have facial dysmorphisms. These individuals presented with obvious mandibular retrusion 9, 10, which is consistent with the zebrafish and mouse phenotypes we observed in this study.

In summary, we reported a novel interaction between the RhoGEF Trio and Myh9. Using Trio knockdown zebrafish and mouse models, we found that Trio might engage in interactions with Myh9 to disturb the migration of NCCs and lead to abnormal osteogenic differentiation, resulting in craniofacial anomalies. Our results, combined with previous findings, revealed that the regulation of Trio during embryogenesis is critical for craniofacial development and may provide diagnostic and therapeutic targets for these genetic defects.

## Methods

### Animals

*Wnt1-Cre* and *Trio^fl/fl^* mice used in this study were obtained from the Model Animal Research Center of Nanjing University, Nanjing, China (MARC). Breeding of the mice has been reported 2-4. To generate NCC-specific knockout mice, we generated a *Wnt1-Cre;Trio^fl/fl^* knockout mouse model by crossing a floxed Trio allele with a *Wnt1-Cre* driver and confirmed the genotype of *Wnt1-cre;Trio^fl/fl^* mice (CKO) using PCR. *Wnt1-Cre* and *Trio^fl/fl^* mice were used as controls (WT) and *Wnt1-Cre;Trio ^fl/fl^* as CKO. Wild type and Tg(*sox10: egfp*) zebrafish were raised as previously described 61 on a 14 h/10 h light/dark cycle at 28.5 ºC in the zebrafish facility of the Model Animal Research Center, Nanjing University. All animals were handled with the approval of the Ethics Committee of the Stomatological School of Nanjing Medical University. All experiments were performed in conformity to the guidance of the Animal Care Committee of Nanjing Medical University.

### Microinjection of morpholino and mRNA

Morpholino (MO) was obtained from Gene Tools (Philomath, USA). A translation-blocking MO targeting zebrafish *trio* with the sequence of 5'-TACTCATCCTCGGATTCAATGGTTC-3' was used. As a specificity verifying control, an additional morpholino was designed with the sequence of 5'- TACTGATGCTCCGATTGAATCGTTC-3'. For non-specific control embryos, a standard control MO was used: 5'-CTAAAAGCA-GCAGGAGGCGATTCAT-3'. For *myh9* knockdown, a translation-blocking MO against zebrafish *myh9* was used as previously described 62. All MO injection was performed at the 1-2 cell stage with a concentration of 2 ng/μL for later experiments. The sequence of Human ca-Rac1 G12V and Human ca-Cdc42 G12V were obtained from the Guthrie cDNA Resource Center (Sayre, PA) 63. The sequence of zebrafish *myh9* was obtained from NCBI Gene Database. Then, the ca-Rac1 G12V, ca-Cdc42 G12V cDNA and *myh9* cDNA were cloned and ligated into the pXT7 plasmid. Later, plasmids were linearized by BamHI and transcribed with the mMESSAGE mMACHINE T7 kit (Ambion, #AM1344). The ca-Rac1 mRNA and ca-Cdc42 mRNA were injected into one-cell stage zebrafish embryos at 10 pg/embryo, the myh9 mRNA was injected at 50 pg/embryo.

### Cartilage Staining and Analysis

For cartilage staining, 120 hpf zebrafish embryos were fixed in freshly prepared 4% paraformaldehyde (PFA) overnight at 4 ºC and stained with Alcian blue (Sigma, #A5268) 64, 65. The samples were rinsed with a KOH/glycerol series until they were clear and finally maintained in 100% glycerol. The key length was measured as described previously 61, 66. Line A served as a baseline for later measurements. Line B represented the distance from line A to the anterior of the Meckel's cartilage. The ratio of line B divided by line A (B/A) between *con* MO and *trio* MO embryos was quantified.

### Whole-mount *In Situ* Hybridization

Whole-mount *in situ* hybridization in zebrafish was performed as previous study 67, 68. Digoxigenin-labelled antisense RNA probes were produced with a DIG RNA labeling kit (Roche, #11175025910). The following probes were used (forward/reverse): *trio* (5'-TACCTGTCCACACACACCT-3'/5'-GGTACGATGAGATGGAAT-3'),* foxd3* (5'-CAAAGCATGTGTCATCTTG-3'/5'-TGAGAATGTCCGGCTGAT-3'), *crestin* (5ʹ-TGCCCTGGAGACGAAACA-3ʹ/5ʹ-CCCACTTCCGATCTGCTT-3ʹ). The whole-mount *in situ* hybridization in mice was performed according to the protocol of the ISH kit (Boster Bio, #MK1031). The probe of *trio* was used (forward/reverse): 5'-GTCCTTAAGGCATCCAGTATC-3'/5'-CAAGGCCTCTTCAAGGTTATT-3'). Briefly, tissue samples were digested with pepsin, then incubated with pre-hybridization solution, followed by hybridizing with digoxigenin-labelled oligo-nucleotide probes overnight. On the next day, the samples were washed with SSC (NaCl+C_6_H_5_O_7_Na_3_·2 H_2_O) and blocked with blocking reagent at 37 ºC, then incubated with alkaline phosphatase-labelled anti-digoxin reagent. Finally, the samples were stained with BCIP/NBT and imaged by upright microscope (Leica Microsystems, Ontario, Canada).

### Time-lapse imaging

Tg(*sox10: egfp*) embryos were used to study NCCs migration *in vivo* 19. Tg(*sox10: egfp*) embryos were injected with *con* MO, *trio* MO, *myh9* MO, *trio* MO+ *myh9* mRNA, *trio* MO+ca-Rac1/ca-Cdc42 mRNA, *myh9* MO+ca-Rac1/ca-Cdc42 mRNA. At 12-16 SS, 21-27 hpf, the embryos were embedded in 1.5% low-melting agarose gel and adjusted in a position with its dorsum and flank upward. Compound microscopes (Leica DM5500 and Zeiss LSM710) were used for live-imaging. Images were captured every 5 min for 7 h.

### Mice skeletal staining

The staining was conducted according to the modified procedures 69. Mutant mice and control mice were fixed in 95% ethanol for at least 1-3 days. Before staining we removed skin, and viscera, particularly the liver, kidneys, and gut. Next, embryos were added to Alcian blue solution and left for 1-2 days. After rehydrating through an ETOH series and H_2_O, 1% KOH solution was used to make them clear. Then, embryos were added to Alizarin red S/KOH solution. Eventually, the specimens were rinsed in 1% KOH and treated with a KOH/glycerol series, and stored in glycerol.

### Micro-CT Analysis

WT and CKO mice at P21 were sacrificed and soft tissues were dislodged. The skulls and femurs were first fixed in 4% PFA and then scanned using a Micro-CT system 70. Reconstruction and analysis were performed using NRecon v1.6 and CTAn v1.13.8.1 software.

### Histology and Immunostaining

Slice samples for histological analysis were collected at a scheduled time and fixed overnight in 4% paraformaldehyde. WT and CKO mice were collected at E9.5, E10.5, E14.5, E15.5, E17.5, P1, and P14. Human teeth were acquired from the extracted teeth of patients treated at the Oral Surgery Department of the Jiangsu Provincial Stomatological Hospital (Nanjing, China). The written informed consent was obtained from the patients. Samples were then dehydrated, paraffin-embedded, and sectioned at 5 μm. For whole-mount immunostaining, Tg(*sox10:egfp*) zebrafish embryos were collected at 10 SS, 12 SS. WT and CKO mice were collected at E9.5 and E10.5. Embryos were collected, fixed, rehydrated, and stained as previously 71. Von Kossa (1% silver nitrate) and total collagen staining (1% Sirius Red) were used to identify the mineralized bone. Immunohistochemical and immunofluorescence staining 72, 73 was used to detect the phenotype differences and the expression of relevant molecules respectively. The following antibodies were used: anti-Trio (1:200, Santa Cruz, sc-28564), anti-Collagen I (1:300, Abcam, #ab21286), Sox-9 (1:100, Santa Cruz, sc-166505). TUNEL staining was performed using the *In Situ* Cell Death Kit (Roche) to assess cell apoptosis. Phosphohistone-H3 immunohistochemistry (Upstate) was performed to detect cell proliferation using p-Histone H3 (Ser10)-R (1:100, Santa Cruz Biotechnology, A2971). Nuclei were stained with 4′,6-diamidino-2-phenylindole (DAPI) at 1:1000. The images were captured under a laser confocal microscope (Zeiss LSM710) with 488 nm and 594 nm laser lines.

### Culture of NCCs

We harvested embryos of mice at gestational day E9.5 71, removed deciduas, and extracted the first branchial arch into PBS with microscopic instruments, then dissociated in 0.25% trypsin-ethylenediaminetetraacetic acid (EDTA, Gibco, #25200056) for 30 min at 37 ºC. The disassociated cells were subsequently plated into large dishes with F12/DMEM (1:1) medium supplemented with 10% FBS, 1% penicillin-streptomycin, 1% L-Glutamate (LEAGENE, #CM0223), 1% Non-Essential Amino Acids (NEAA, Gibco, #11140050) and 0.1% leukemia inhibitory factor (LIF, ESGRO, #ESG1106). Media were changed every three days.

### Lentiviral transfection

One day before lentiviral infection, NCCs were seeded in large dishes at a density of 1 × 10^8^ cells per dish and cultured overnight. At the time when cells grew to 70% confluence, the media were replaced with medium without penicillin-streptomycin containing 5 μg/mL polybrene, at the same time Lentiviral particles encoding shRNA targeting the Trio gene (shTrio 5ʹ-CCAGCTAACTCCCGAGTTT-3ʹ) and Myh9 gene (shMyh9 5ʹ-GUGCCAACAUUGAGACUUATT-3ʹ) or a negative control shRNA (shCtrl 5ʹ-TTCTCCGAACGTGTCACGT-3ʹ), Multiplicity of infection (MOI) = 50. The next day, the supernatant was removed and a fresh medium was added. The fluorescence microscopy (Leica Microsystems, Ontario, Canada) was used to measure the efficiency of the lentivirus infection 72 h later.

### Quantitative reverse transcription PCR for mRNA analysis (qRT-PCR)

Total cell RNA was extracted using an RNA isolation kit (BioTeke, Beijing, China). Complementary deoxyribonucleic acid (cDNA) was generated using PrimeScript RT reagent kit (TaKaRa, #RR047A). quantitative reverse transcription PCR (qRT-PCR) reaction was performed using ChamQ SYBR qPCR Master Mix (Vazyme, Nanjing, China) on the ABI-7300 Real-Time PCR System (Applied Biosystems, CA, USA). The primers used are listed in Table S1. Data were analyzed using the 2^-∆∆CT^ method.

### Cell proliferation assay

To evaluate the effects of Trio on NCC proliferation, the Cell Counting Kit-8 (CCK-8; Dojindo Kagaku Co, Kumamoto, Japan) was used. shCtrl and shTrio NCCs were seeded at 3 × 10^3^ cells per well in 96-well plates, incubated at 5% CO_2_ 37 ºC for 12 h (day 0). CCK-8 reagent mixed with fresh medium 1:10 was added to each well at different time points (day 0, 1, 3, 5, 7). After 1.5 h of incubation, optical density (OD) was detected at 450 nm using a microplate reader.

### Flow cytometry assay

NCCs transfected with shCtrl or shTrio were assessed for cell cycle and apoptosis by flow cytometry. The cell cycle was detected by staining with propidium iodide (PI), followed by using flow cytometry (FACSCalibur, BD Biosciences). Cell apoptosis was detected by staining with Annexin-V-APC (Annexin-V-Allophycocyanin) and 7AAD (7-Aminoactinomycin) stains (BD Pharmingen, Franklin Lakes, USA). The analysis was performed by FACS can cytometry (Becton-Dickinson, SanJose, CA, USA). In this study, the apoptotic cells were considered as the early apoptotic cells (B2: Annexin V+/7AAD- staining) and the late apoptotic cells (B4: Annexin V+/7AAD+ staining). The data were analyzed by Flowjo V7.

### Cell migration assay

The shCtrl and shTrio cells were seeded at a density of 8 × 10^5^ cells per well in 6-well plates. When cells grew to 90% confluence, a pipette tip was used to scratch a wound at the bottom of the wells, and the floating cells were removed by washing the cells once with PBS. Wound healing was captured at 0 h, 12 h and 24 h using a Leica DMIRE 2 microscope in phase contrast mode and Leica FW 4000 software.

### Transwell assay

The transwell assay was used after lentivirus transfection to assess the effect on migration. Cells were seeded on the upper chamber and 600 uL of media was added into the lower chamber. After incubation for 12 h and 24 h, the migrated cells were fixed in 4% PFA and stained with 0.1% methylrosanilinium chloride for 30 min, the cells were observed with the upright microscope (Leica Microsystems, Ontario, Canada).

### Osteogenic differentiation Assay

NCCs were incubated in osteogenic medium for 5 days or 14 days, fixed in 4% PFA, and the ALP activity kit (Beyotime Institute of Biotechnology, Shanghai, China) was used to measure the Alkaline Phosphatase (ALP) activity and 2% Alizarin red was used to stain the mineralization nodules.

### Immunofluorescence

Cells were plated onto a 48-well plate at 2 × 10^4^ cells/well and cultured for 24 h. Then, fixed with 4% paraformaldehyde for 30 min at room temperature and subsequently permeabilized with 0.5% Triton X-100 for 20 min at room temperature. The slides were then treated with goat serum for 30 min at room temperature, incubated with the following primary antibodies: Trio (1:300, Abcam), Myh9 (1:100, Proteintech), β-tubulin (1:100, Proteintech), Collagen I (1:300, Abcam), GM130 (1:100, Abcam), Pax7 (1:1000, Proteintech), Snai2 (1:1000, Proteintech), Foxd3 (1:1000, R&D Systems), Sox9 (1:1000, Cell Signaling Technology) on the slides at 4 ºC overnight. The coverslips were treated with fluorescence-conjugated secondary antibodies and phalloidin (Cytoskeleton) diluted in the blocking solution for 1 h. Then, the cells were washed with PBS and the coverslips were mounted with DAPI before imaging the cells for 90 seconds at room temperature. Photos were taken by laser confocal scanning microscopy (Carl Zeiss, Heidenheim, Germany) and Leica DM 4000 Fluorescence System.

### Western blot

The proteins isolated from cells using RIPA buffer and PMSF were centrifuged at 12,000 rpm for 15 min at 4 ºC, boiled for 5 min. Equal levels of proteins were separated into 10% or 12% SDS-PAGE, a 6% SDS-PAGE used to detect Trio, and then transferred into PVDF membranes, blocked with 5% non-fat milk for 2 h and then incubated with primary antibodies against the following epitopes: Trio (1:200, Abcam, USA), Myh9 (1:100, Proteintech), Rac1 (1:500, Cytoskeleton), Cdc42 (1:250, Cytoskeleton), PAX7 (1:1000, Proteintech), SNAI2 (1:1000, Proteintech), FOXD3 (1:1000, R&D Systems), SOX9 (1:1000, Cell Signaling Technology), GAPDH (1:8000, Bioworld), Histone H3 (1:1000, Cell Signaling Technology) overnight at 4 ºC. The membranes were washed with Tris buffered saline (TBST) 3 times and then incubated with secondary antibody (1:8000, KPL, USA) for 1 h at room temperature, washed with TBST 3 times again. The enhanced chemiluminescence (Thermo Fisher Scientific, Rockford, IL) was used to visualize proteins. Proteins were imaged with Chemiluminescence gel imaging system (Tonon 5200) and semi quantified by Image J software.

### Isobaric tags for relative and absolute quantitation (iTRAQ)

Total protein extracted from NCCs transfected shCtrl and shTrio were digested overnight at 37 ºC with trypsin. After trypsin digestion, peptides were processed according to the manufacturer's protocol for 6-plex iTRAQ reagents. Then the iTRAQ labeled peptides were fractionated and dried using vacuum centrifugation. Finally, the mixed fractions were subjected to LC-MS/MS analysis.

### Rac1 and Cdc42 Activation assay

The activities of Rac1 and Cdc42 were measured using Rac1 and Cdc42 pull-down Activation Assay Biochem Kit (BK035/BK036, Cytoskeleton Inc) according to the manufacturer's instructions. Briefly, the active form of Rac1 and Cdc42 were selectively pulled down from the lysate of NCCs transfected with shCtrl and shTrio with the PAK-PBD beads. Subsequently, the bound GTP-Rho was detected by Western blot analysis with anti-Rac1 antibody (1:500) and anti-Cdc42 antibody (1:250). For the Rac1-GTP inhibitor NSC23766 (Selleck) transfection, the cells were incubated in the medium containing 50 μM NSC23766 for 24 h.

### GST-pull-down assay

To verify the interaction of Trio with Myh9, pull-down assay was performed. Mice Myh9 and Trio were inserted into pCZN-1 vector, pCZN1-Myh9 and pCZN1-Trio were transfected into E coli Arctic Express. Myh9-head and Trio-GEFD1 were purified from E coli Arctic Express cells then run on a 12% SDS-PAGE, stained with Coomassie blue to analyze the concentration of the protein. Purified His-Myh9 was mixed with purified GST, GST-Trio in a 1.5mL tube, and incubated at 4 ºC overnight with rotation, followed by centrifugation at 1250 ×g for 5 min and washing 3 times with PBS. Proteins were eluted and next analyzed by Western blot using anti-His (QED, 18814-01) and anti-GST (Sigma, G1160).

### Proximity Ligation Assay (PLA)

The PLA was performed to confirm the interaction of Trio with Myh9 according to the manufacturer's protocol (Sigma Aldrich #DUO92102). In short, NCCs were seeded onto slides in a 24-well plate at a density of 2 × 10^4^ cells/well, washed 2 times with PBS and fixed in 4% PFA for 20-30 min and then permeabilized in 0.5% Triton X-100 for 20 min, washed 2 times with PBS, blocked with blocking reagents for 60 min at 37 ºC. After blocking, cells were incubated with primary antibodies against Trio (1:100, Abcam) and Myh9 (1:100, proteintech) or RAC1 (1:100, Cytoskeleton) and β-catenin (1:100, Cell Signaling Technology) overnight at 4 ºC, followed by corresponding secondary antibodies conjugated with PLA probes for 60 min at 37 ºC. Cells were then incubated with ligation reagent for 30 min and with amplification reagent for 100 min at 37 ºC in dark. Next, cells were washed three times with wash buffer and incubated with a mounting medium with DAPI for 15 min in dark. Finally, Duolink and DAPI signals were detected using confocal microscopy (ZEISS CSM710).

### Cell Polarization Assay

NCCs were seeded on glass coverslips to reach 90% confluence. Followed by starvation in serum-free medium for 24 h, NCCs were wounded with a 10 uL pipette tip and washed by PBS. Subsequently, cells were incubated in a medium with 5% FBS for 24 h, and then fixed in 4% PFA, and incubated with GM130 (#ab2899, Abcam) at 4 ºC overnight. The next day, the cells were incubated with secondary antibodies to recognize GM130 and phalloidin-FITC to recognize F-actin and then incubated with DAPI to detect Nuclei. The cells were then visualized with a confocal microscope (ZEISS CSM710). The position of the Golgi apparatus and nucleus reorientation was measured as previously reported 74. Cdc42 specific activator Bradykinin was purchased by SIGMA, Munich, Germany.

### Dual-luciferase reporter assay

293T cells were seeded in 24-well plates at a density of 3.0 × 104 cells per well. After 24h, they were co-transfected with Pgl3‐basic luciferase reporter vector (Promega, Madison, WI, USA) containing the 3′‐UTR fragment of Foxd3 or Snai2 or Sox9 or Pax7, renilla vector (pRL‐TK; Promega, Madison, WI, USA), negative control siRNA and β-catenin siRNA (GenePharma, Shanghai, China) using Lipofectamine 2000 (Invitrogen). After 48h of transfection, Luciferase activity was measured by the Dual Luciferase Reporter Assay System (Promega, Madison, WI, USA). The ratio of firefly luciferase activity to renilla luciferase activity was calculated for each sample.

### Statistical Analysis

All experiments were carried out at least three times. All data were expressed as the mean standard error of the mean (S.E.M.). The results in the control and experimental groups were analyzed by GraphPad PRISM software (ver.8.3.0, La Jolla, CA). *p* < 0.05 was considered statistically significant.

## Supplementary Material

Supplementary figures.

Supplementary table S1.

Supplementary table S2.

Supplementary table S3.

Supplementary table S4.

Supplementary table S5.

Supplementary movie S1.

## Abbreviations

ALP: alkaline phosphatase
ARS: Alizarin red staining
ca*-*Rac1/ca*-*Cdc42: constitutively active form of Rac1/Cdc42
CCK-8: Cell Counting Kit-8
DAPI: 4, 6-diamidino-2-phenylindole
DEPs: differentially expressed proteins
DVL: Dishevelled
EMT: epithelial to mesenchymal transition
E: embryonic day
P: postnatal day
GO: Gene Ontology
hpf: hours post-fertilization
iTRAQ: isobaric tags for relative and absolute quantification
KEGG: Kyoto Encyclopedia of Genes and Genomes
NCC: neural crest cell
MO: morpholino
PLA: proximity ligation assay
pa1: pharyngeal arch 1
PHH3: phosphohistone H3
PI: propidium iodide
qRT-PCR: Real-time quantitative polymerase chain reaction
SS: somite stage
SCAPs: stem cells of dental papilla
TUNEL: terminal deoxynucleotidyl transferase dUTP nick end labeling
WISH: whole-mount *in situ* hybridization
