# Supplementary material for: Trio cooperates with Myh9 to regulate neural crest-derived craniofacial development
Source: Theranostics. 2021 Feb 25;11(9):4316–34. doi: 10.7150/thno.51745 (PMC7977452; doi:10.7150/thno.51745)
Supplement: Supplementary file 1 — Supplementary figures. [file thnov11p4316s1.pdf]

Supplementary Information

Trio cooperates with Myh9 to regulate neural crest-derived craniofacial development

Shuyu Guo, Li Meng, Haojie Liu, Lichan Yuan, Na Zhao, Jieli Ni, Yang Zhang, Jingjing Ben, Yi-ping Li, and Junqing Ma

Figure S1

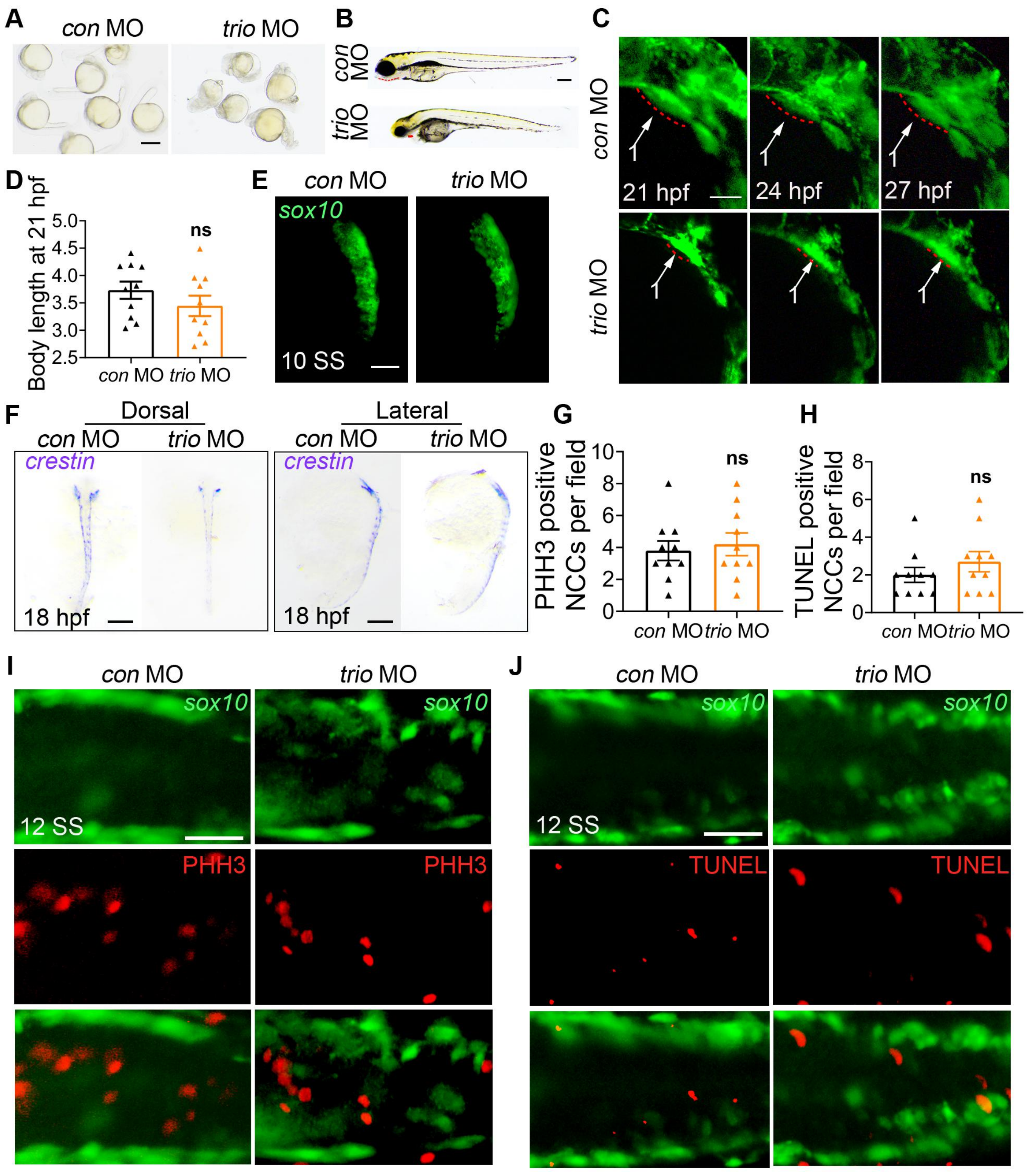

Figure S1: *trio* loss-of-function caused defects in NCC descendant differentiation and migration. (**A**) Represent bright field images of *con* MO embryos and *trio* MO embryos at 24 hpf. Bar = 200 μm. hpf: hours post-fertilization; MO: morpholino. (**B**) Represent bright field images of *con* MO and *trio* MO embryos at 96 hpf. Bar = 100 μm. Red dotted line: mandible. (**C**) Lateral view of time-lapse images of *con* MO and *trio* MO Tg*(sox10:egfp)* embryos at 21, 24, 27 hpf. The red dotted lines outline the first NCC

migratory stream. Bar = 500 μm. (**D**) Quantification of body length of *con* MO and *trio* MO embryos at 21 hpf (n = 10). (**E**) *con* MO and *trio* MO Tg*(sox10:egfp)* zebrafish at 10 SS. SS: somite stage. Bar = 500 μm. (**F**) WISH performed for the NCC migration marker *crestin* in *con* MO and *trio* MO zebrafish embryos. The dorsal and lateral views of 18 hpf embryos show *crestin* expression in NCC migratory streams. Bar = 200 μm. WISH: whole-mount in situ hybridization. (**G, I**) Image and quantitative analysis of PHH3 staining of *con* MO and *trio* MO Tg*(sox10:egfp)* embryos indicate NCC proliferation in the dosal neural tube at 12 SS (n = 10). PHH3: phosphohistone H3. Bar = 500 μm. (**H, J**) Image and quantitative analysis of TUNEL staining of *con* MO and *trio* MO Tg*(sox10:egfp)* embryos indicate NCC apoptosis in the dorsal neural tube at 12SS (n = 10). Bar = 500 μm. TUNEL: terminal deoxynucleotidyl transferase dUTP nick end labeling. All data are represented as mean ± S.E.M (two-tailed t test ns, not significantly).

Figure S2

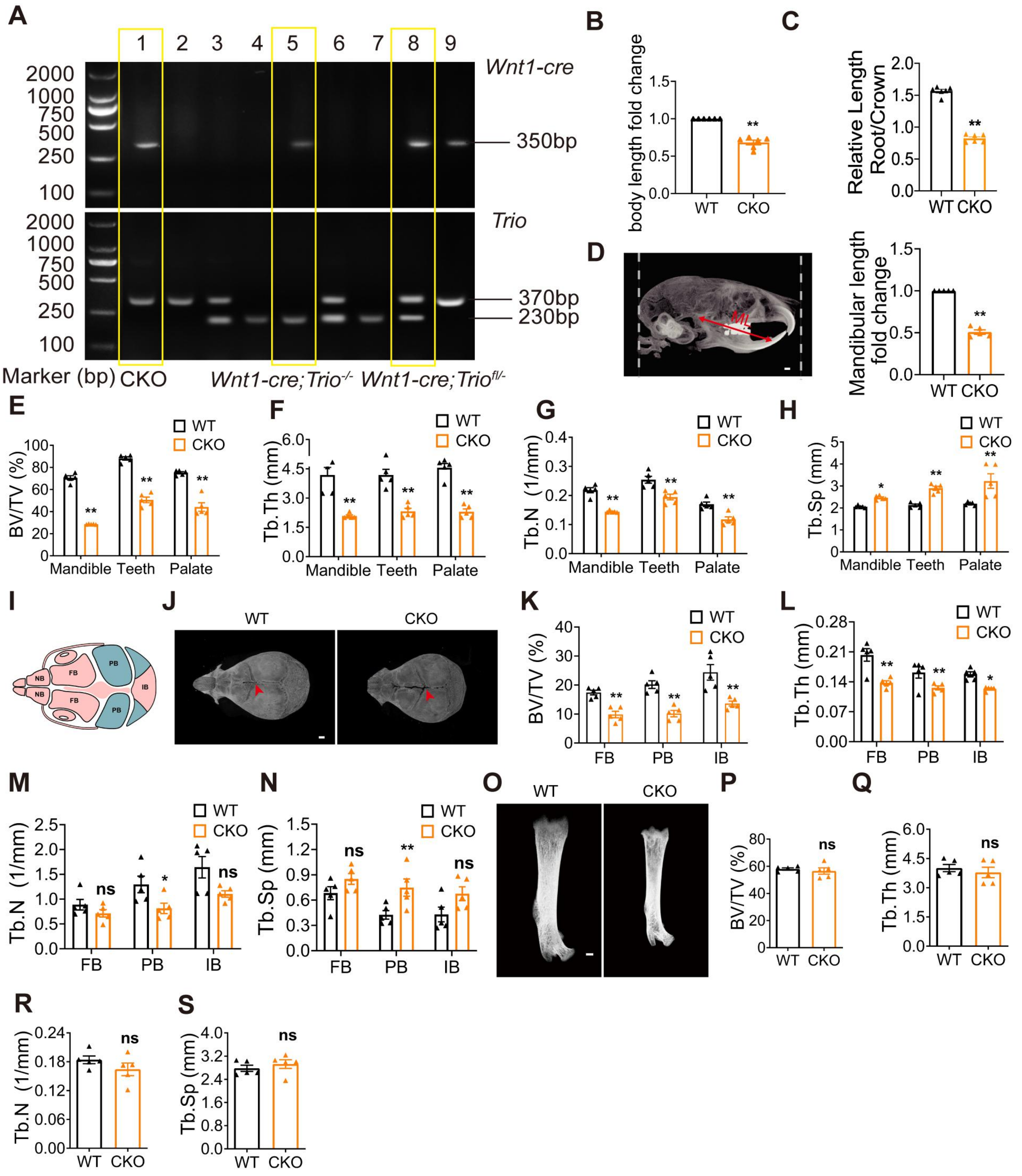

Figure S2: Trio-conditional-knockout mice showed more osteoporotic bone in the skull, but had no change in the femur.

(**A**) Mice DNA genotypes of *Wnt1-cre* and *Trio-flox* analyzed by PCR. Upper gel represented bands of *Wnt1-cre*. The lower gel contained bands of *Trio*. *Wnt1-cre*: 350-bp, *Trio-flox* homozygote ($Trio^{fl/fl}$): 370-bp, *Trio-flox* heterozygote ($Trio^{fl/-}$): 370 and 230-bp, *Trio* wildtype ($Trio^{-/-}$): 230-bp. Lane 1: *Wnt1-cre;*$Trio^{fl/fl}$ = CKO, lane 5: *Wnt1-cre;*$Trio^{-/-}$, lane 8: *Wnt1-cre;*$Trio^{fl/-}$. (**B**) Quantification of body length fold change of WT and CKO mice (n = 5). (**C**) Quantification analysis of relative length root/crown of WT and CKO mice at P21 (n = 6). (**D**) Longitudinal sketch of skull and quantification of mandibular length (ML) (n = 5). Bar = 500 μm. (**E-H**) Quantification of Micro-CT images of the mandible, teeth, palate area obtained from WT and CKO mice at P21. BV/TV: bone volume fraction, Tb.Th: trabecular thickness; Tb.N: trabecular number; Tb.Sp: trabecular separation (n = 5). (**I**) Schematic top view of mice skull. Pink indicated the origin of neural crest and blue indicated the origin of mesoderm. NB: nasal bone; FB: frontal bone; PB: parietal bone; IB: interparietal bone. (**J**) Top scanning of WT and CKO mice skull at P21. CKO skull displayed incomplete closure of cranial surture (red arrow). (**K-N**) Quantification of micro-CT images of frontal bone (FB), parietal bone (PB) and interparietal bone (IB) from WT and CKO mice at P21. (**O**) Longitudinal scanning of femur. Bar = 500 μm. (**P-S**) Quantification of BV/TV, Tb.Th, Tb.N, Tb.Sp of femur (n = 5).All data are represented as mean ± S.E.M (two-tailed t test $*p < 0.05$, $**p < 0.01$, ns, not significantly).

Figure S3

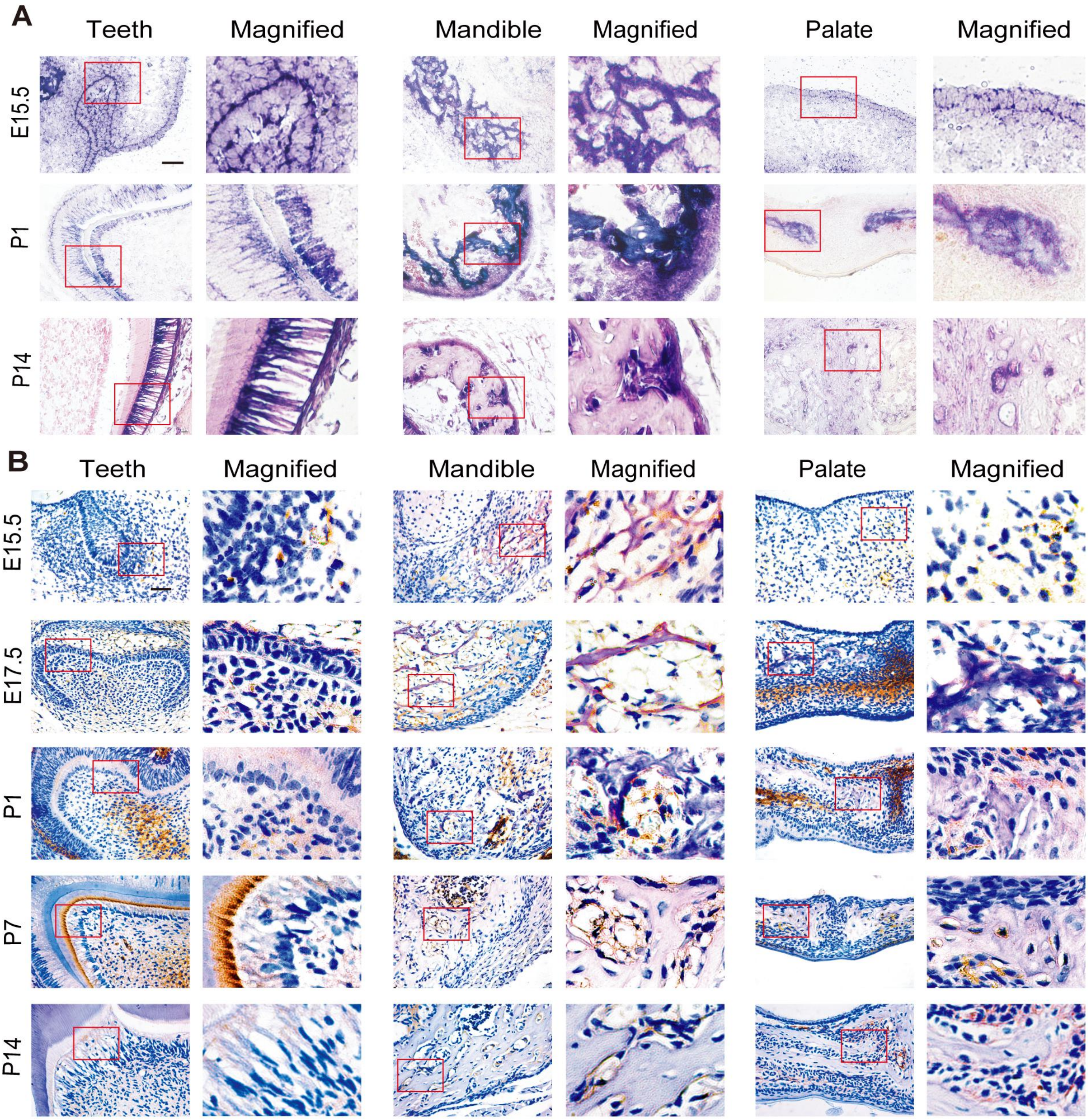

Figure S3: Trio showed expression in teeth, mandible and palate of mice.

(**A**) WISH of Trio expression of mice at E15.5, P1 and P14. Bar = 100 μm. Following panels showed higher magnifications of boxed areas. (**B**) Immunohistochemical staining for Trio expression of mice at E15.5, E17.5, P1, P7 and P14. Bar = 100 μm. Following panels showed higher magnifications of boxed areas.

Figure S4

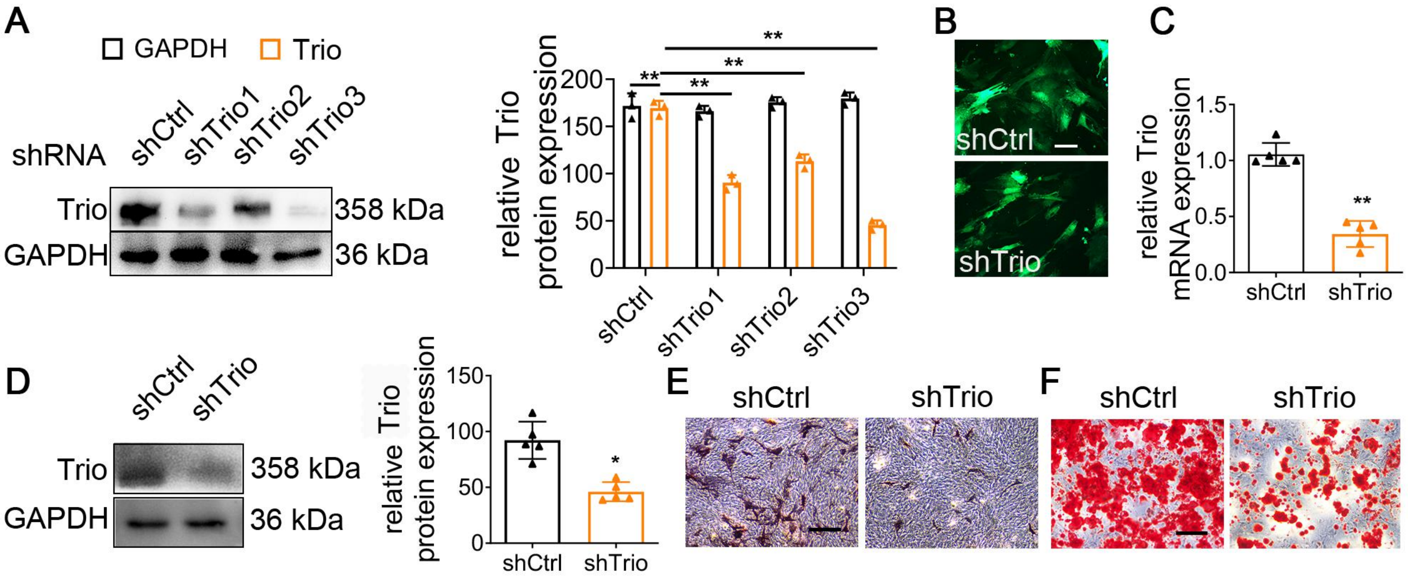

Figure S4: Knockdown of Trio in NCCs influenced cell proliferation, apoptosis and osteogenesis differentiation *in vitro.*

(**A**) Lentiviral particles encoding shRNA-3 targeting the Trio gene (shTrio3) was selected for its high Trio knockdown efficiency by western blot analysis. Quantitative analysis of western blot bands followed (n = 3). (**B**) Fluorescence microscopy images proved shTrio lentivirus transfection efficiency of NCCs. Bar = 100 μm. (**C**) qRT-PCR analysis performed to detect Trio mRNA expression in shCtrl and shTrio NCCs (n = 5). (**D**) Western blot conducted to test the Trio knockdown efficiency after shTrio transfection. Quantitative analysis of western blot bands followed (n = 5). (**E**) Alkaline phosphatase (ALP) staining of NCCs. Bar = 100 μm. (**F**) Alizarin red staining (ARS) of NCCs. Bar = 100 μm. All data represent mean ± S.E.M (two-tailed t test $*p < 0.05$, $**p < 0.01$, ns, not significantly).

Figure S5

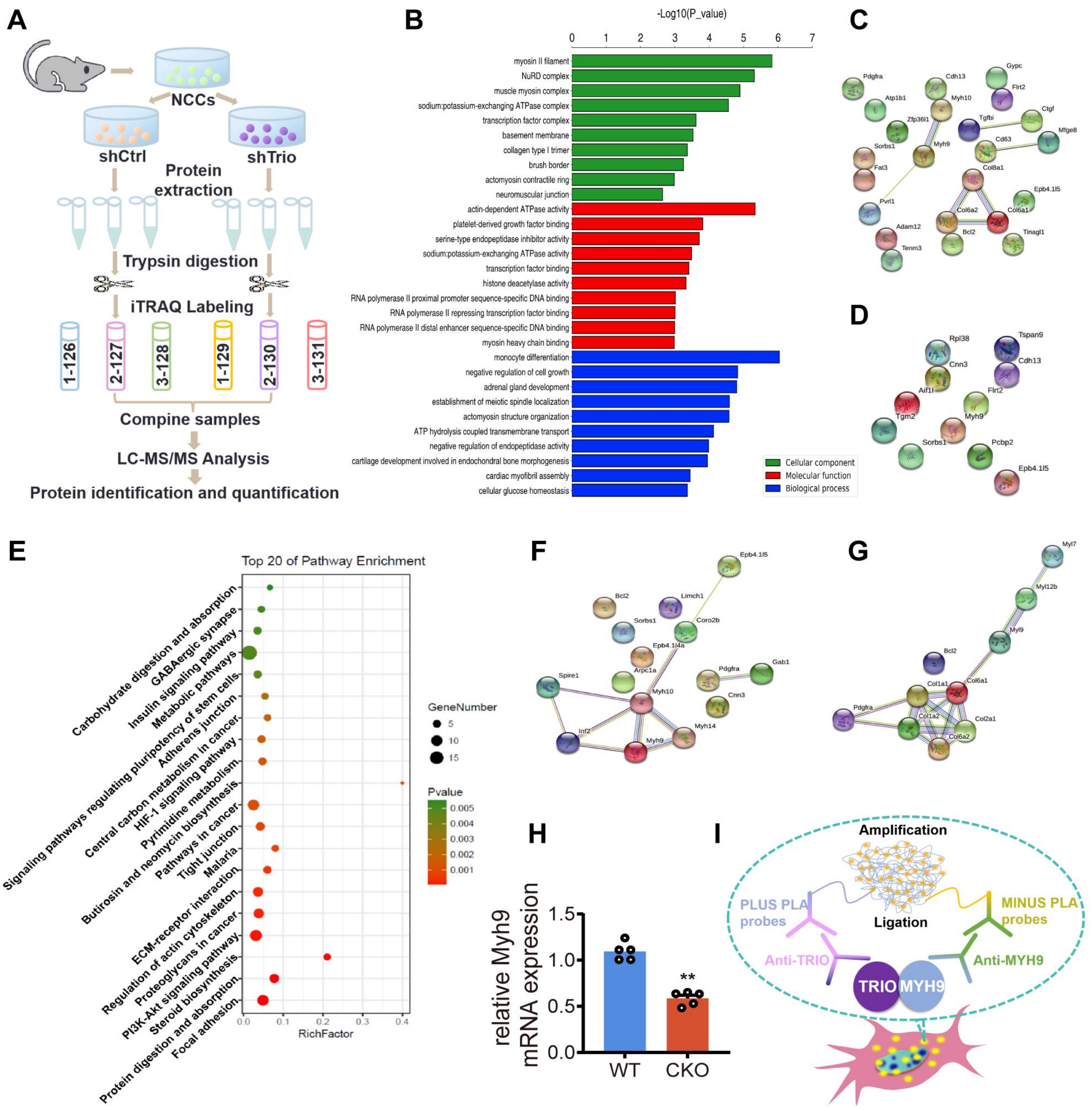

Figure S5: Selection for Myh9 from 297 DEPs in iTRAQ by bioinformatic analysis.

(**A**) Schematic outline of the sample preparation and iTRAQ protein analysis in shCtrl and shTrio NCCs. iTRAQ, isobaric tags for relative and absolute quantitation. (**B**) GO enrichment analysis of common DEPs between shCtrl and shTrio group. The vertical coordinate represented categories sorted according to p-value from top to bottom. The more significant items were to the top. (**C**) main GO clusters within the biological process (BP) involving in cell. adhesion. (**D**) Main GO clusters within the cell component (CC) involving in focal adhesion. (**E**) Top 20 enrichment KEGG pathways associated with DEPs. The pathway ranking in this diagram is in order of smaller *P*-value and bigger

gene number from top to bottom.(**F**) Main GO clusters within the cell component (CC) involving in actin-cytoskeleton. (**G**) Main KEGG clusters involving in focal adhesion. (**H**) qRT-PCR analysis performed to detect Myh9 mRNA expression in the first branchial arch of WT and CKO embryos at E10.5 (n = 5). (**I**) Schematic of the proximity ligation assay (PLA) in NCCs. All data represent mean ± S.E.M (two-tailed t test $**p < 0.01$).

Figure S6

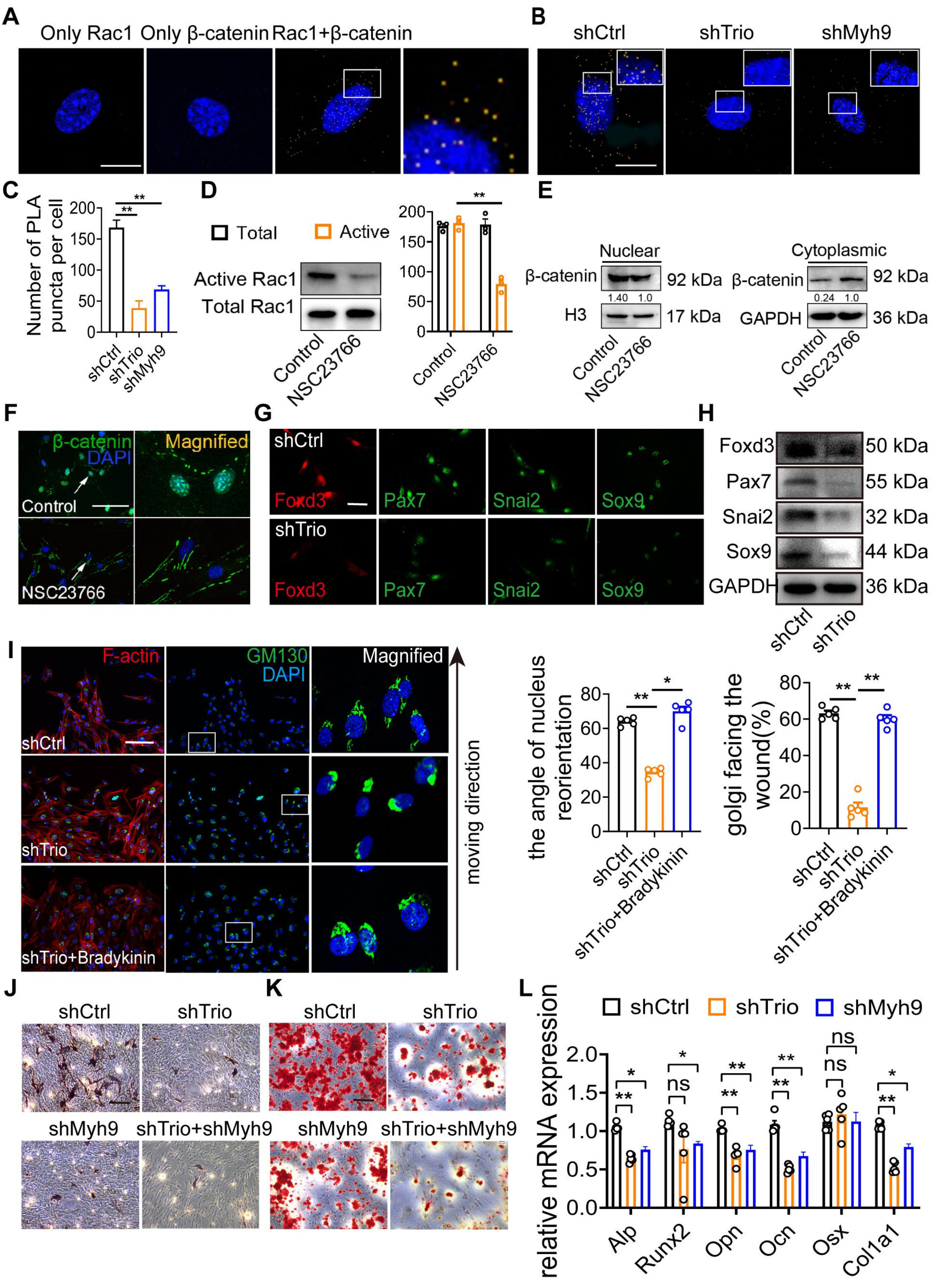
A
Only Rac1
Only β-catenin
Rac1+β-catenin
B
shCtrl
shTrio
shMyh9
C
Number of PLA puncta per cell
D
Total
Active
Active Rac1
Total Rac1
Control
NSC23766
E
Nuclear
β-catenin
92 kDa
1.40
1.0
H3
17 kDa
Cytoplasmic
0.24
GAPDH
36 kDa
F
β-catenin
DAPI
Magnified
Control
NSC23766
G
Foxd3
Pax7
Snai2
Sox9
H
50 kDa
55 kDa
32 kDa
44 kDa
I
F-actin
GM130
shTrio+Bradykinin
moving direction
the angle of nucleus reorientation
golgi facing the wound(%)
J
shTrio+shMyh9
K
L
relative mRNA expression
Alp
Runx2
Opn
Ocn
Osx
Col1a1
ns

Figure S6: Myh9 assists Trio in regulation of NCC migration via Rac1 and Cdc42 GTPase activation.

(**A**) PLA detection and visualization of Rac1 and β-catenin in NCCs. Blue: DAPI nuclear staining. Yellow spots: PLA signals. Bar = 400 μm. (**B**) PLA detection and visualization of Rac1 and β-catenin in the shTrio and shMyh9 NCC groups. Blue: DAPI nuclear staining. Yellow spots: PLA signals. Bar = 400 μm. (**C**) Quantification of PLA in (**B**) (n = 3). (**D**)Image and quantification of Rac1 pull-down activation in the Control and NSC23766 NCC groups (n = 3). (**E**) Western blot of β-catenin in the nucleus and cytoplasm in the Control and NSC23766 NCC groups. (**F**) Immunostaining of β-catenin (green) and DAPI (blue) for nuclear β-catenin expression indicated by a white arrow in the Control and NSC23766 NCCs. Bar = 100 μm. (**G**) Immunofluorescence staining for markers of migratory NCCs (Foxd3, Pax7, Snai2 and Sox9). Bar = 200 μm. (**H**) Western blot of markers of migratory NCCs (Foxd3, Pax7, Snai2 and Sox9) in shCtrl and shTrio cells. (**I**) Immunofluorescence staining of the Golgi body marker GM130 and F-actin for cell polarization assay. Nuclei were counterstained with DAPI. Quantification analysis of percentage of the angle of nucleus reorientation and Golgi facing the wound (n = 5). Bar = 100 μm. (**J, K**) ALP and ARS staining of shCtrl, shTrio, shMyh9 and shTrio + shMyh9 NCCs. Bar = 100 μm. (**L**) qRT-PCR of marker genes in osteogenic differentiation (Alp, Runx2, Opn, Ocn, Osx, Col1a1) expression (n = 5). All data represent mean ± S.E.M (two-tailed t test $*p < 0.05$, $**p < 0.01$, ns, not significantly).

Figure S7

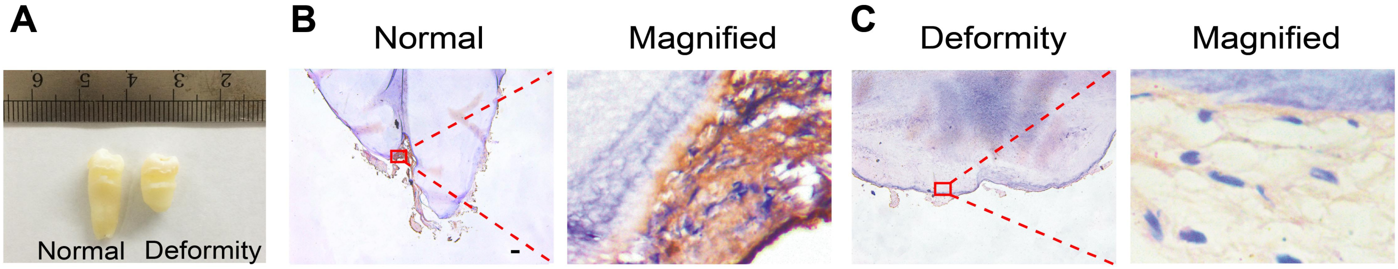

Figure S7: SCAPs in teeth with short roots showed less Trio expression.

(**A**) General view of normal and deformed teeth of human. (**B, C**) Immunohistochemistry for Trio for human stem cells of dental papilla (SCAPs) of normal and deformed teeth. Brown staining indicated anti-Trio immunoreactivity. Teeth with short roots expressed decreased Trio. Bar = 100 µm.
